# Landscape resistance constrains hybridization across contact zones in a reproductively and morphologically polymorphic salamander

**DOI:** 10.1038/s41598-021-88349-7

**Published:** 2021-04-29

**Authors:** Guillermo Velo-Antón, André Lourenço, Pedro Galán, Alfredo Nicieza, Pedro Tarroso

**Affiliations:** 1 CIBIO/InBIO, Centro de Investigação em Biodiversidade e Recursos Genéticos da Universidade do Porto, Instituto de Ciências Agrárias de Vairão. R. Padre Armando Quintas, 4485-661 Vairão, Portugal; 2 Universidade de Vigo, Grupo de Ecoloxía Animal, Departamento de Ecoloxía e Bioloxía Animal, Torre Cacti (Lab 97), 36310 Vigo, Spain; 3 Departamento de Biologia da Faculdade de Ciências, Universidade do Porto. Rua Campo Alegre, 4169-007 Porto, Portugal; 4 Grupo de Investigación en Bioloxía Evolutiva (GIBE), Departamento de Bioloxía, Facultade de Ciencias, Universidade da Coruña, Campus da Zapateira, s/n, 15071 A Coruña, Spain; 5 Departamento de Biologıa de Organismos y Sistemas, Universidad de Oviedo, Oviedo, Spain; 6 Unidad Mixta de Investigacion en Biodiversidad (UMIB), CSIC-Universidad de Oviedo-Principado de Asturias, Mieres, Spain

**Keywords:** Genetic hybridization, Herpetology, Molecular ecology

## Abstract

Explicitly accounting for phenotypic differentiation together with environmental heterogeneity is crucial to understand the evolutionary dynamics in hybrid zones. Species showing intra-specific variation in phenotypic traits that meet across environmentally heterogeneous regions constitute excellent natural settings to study the role of phenotypic differentiation and environmental factors in shaping the spatial extent and patterns of admixture in hybrid zones. We studied three environmentally distinct contact zones where morphologically and reproductively divergent subspecies of *Salamandra salamandra* co-occur: the pueriparous *S. s. bernardezi* that is mostly parapatric to its three larviparous subspecies neighbours. We used a landscape genetics framework to: (i) characterise the spatial location and extent of each contact zone; (ii) assess patterns of introgression and hybridization between subspecies pairs; and (iii) examine the role of environmental heterogeneity in the evolutionary dynamics of hybrid zones. We found high levels of introgression between parity modes, and between distinct phenotypes, thus demonstrating the evolution to pueriparity alone or morphological differentiation do not lead to reproductive isolation between these highly divergent *S. salamandra* morphotypes. However, we detected substantial variation in patterns of hybridization across contact zones, being lower in the contact zone located on a topographically complex area. We highlight the importance of accounting for spatial environmental heterogeneity when studying evolutionary dynamics of hybrid zones.

## Introduction

The spatial extent of hybrid zones (i.e. areas where gene flow between lineages occurs), and the degree of admixture between taxa in these areas are influenced by endogenous (genetic factors) and exogenous (environmental factors) selection, but also through extrinsic neutral processes (e.g. landscape barriers to gene flow). It has been shown that genomic incompatibilities between parent taxa at prezygotic (e.g. reduced fertilization success) and/or postzygotic level (e.g. reduce hybrid fitness) influence evolutionary dynamics in hybrid zones^1–4^. Environmental variation (i.e. isolation by environment, IBE) may also promote selection for or against hybrids^5,6^, or ecological segregation of parent taxa^7^, while landscape features (e.g. rivers, mountains, roads) can act as barriers to dispersal and gene flow (i.e. isolation by resistance, IBR) across heterogeneous landscapes^8–10^. Additionally, the historical context underlying the formation and maintenance of hybrid zones can also affect the extent to which contemporary gene flow occurs between species, with past demography and age of contact zones comprising relevant factors^11–13^.

Contact zones involving intraspecific evolutionary units (e.g. subspecies) often resulted from allopatric differentiation of neighbouring lineages in distinct refugia during unfavourable climatic periods of the Pleistocene, followed by population expansions during climatically favourable periods^14^. These lineages seldom achieve reproductive isolation and continue to hybridize in secondary contact zones^15^. The evolutionary fate of hybrid zones depends on the strength of barriers to gene flow between diverged lineages. On the one hand, secondary contact zones can result in extensive admixture of parental genotypes or lineage fusion when there are not effective barriers to gene flow^16^. On the other hand, selection against hybrids can favour the evolution of barriers to gene flow and ultimately lead to reproductive isolation and speciation^17^. However, an intermediate scenario commonly found in secondary contact zones involves the formation of tension zones often characterized by an equilibrium between dispersal and selection^4,18^.

Phenotypes are targets of selection, playing a major role in species’ performance (e.g. dispersal potential), recognition, and diversification^19^, ultimately dictating the extent and population dynamics of hybrid zones through processes such as assortative mating and overdominance^4,20,21^. Thus, gene flow between subspecies or lineages occurring in secondary contact zones is also determined by their genomic and phenotypic differentiation^11,22–24^. Accounting for this phenotypic differentiation together with the bioclimatic gradients and landscape heterogeneity across contact zones is crucial to understanding the evolution of phenotypic traits and patterns of introgression. This is especially relevant under a scenario of pronounced climate and landscape changes driven by anthropogenic activities because they can alter the evolutionary trajectories of hybridizing taxa^25^ and the fate of evolutionary significant units^26^.

**Figure 1:**
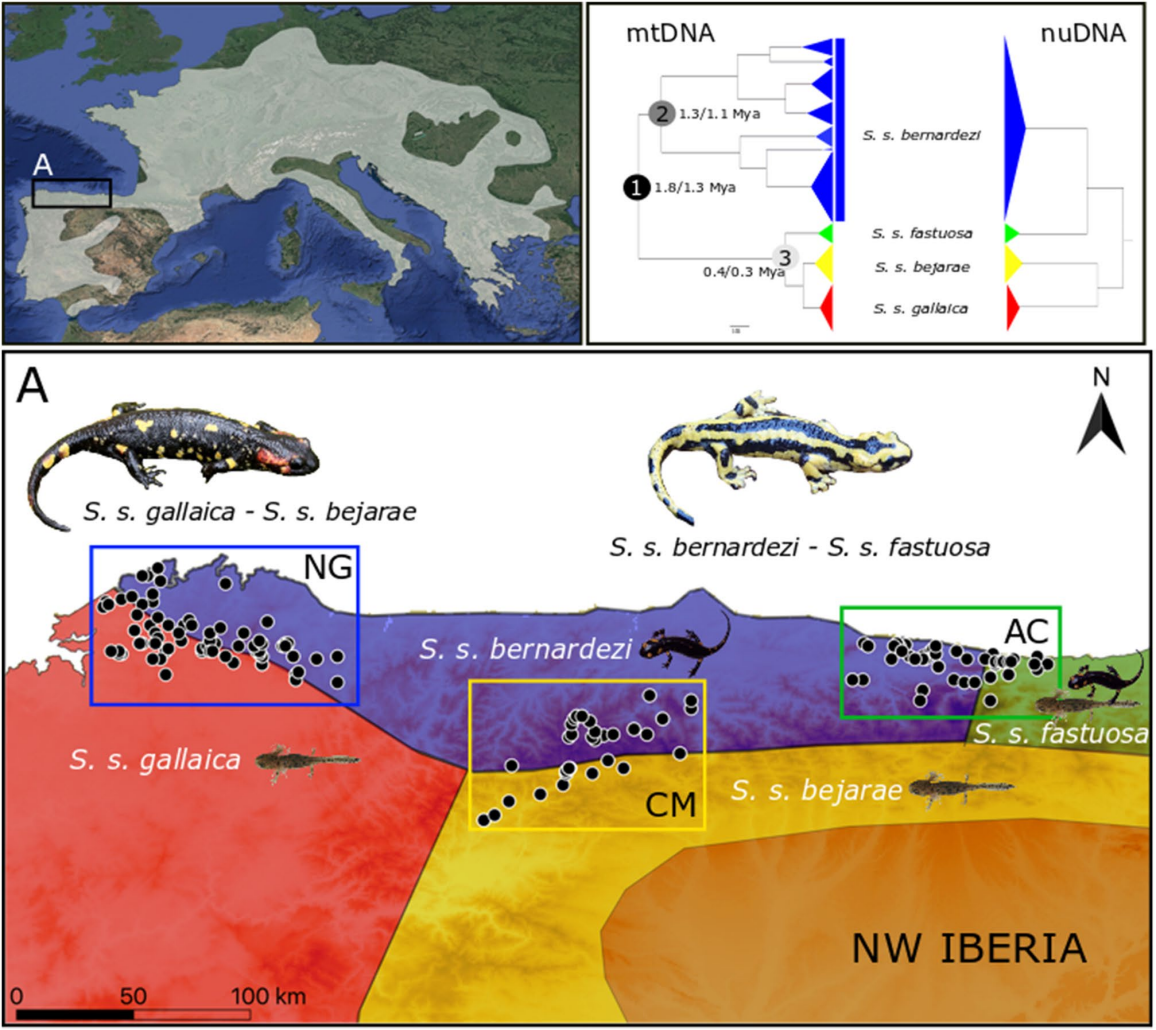
Upper left: distribution of the fire salamander, *Salamandra salamandra*, with the study area denoted with an inset (**A**). Upper right: mtDNA and nuDNA trees to show subspecies relationships and approximate dates of major splits. The mtDNA tree was generated with cytochrome b sequences from this study while the nuDNA tree is adapted from the topology obtained with phylogenomic data in Burgon et al.^30^. Bottom: sampling sites across each of the studied contact zone: North Galicia (NG), Cantabrian Mountains (CM) and Asturias-Cantabria border (AC). Adult salamander images represent the two morphotypes, while the reproductive mode of each studied subspecies (larviparous, pueriparous or both) is represented on the polygons depicting each subspecies’ distribution. The figure was created with QGIS software (version 3.2; https://qgis.org/en/site/). The satellite image was retrieved from Google Earth using the QuickMapServices plugin.

The fire salamander, *Salamandra salamandra* (Linnaeus, 1758), is widely distributed in Europe and offers an exceptional model system for studying neutral and adaptive processes, particularly in the Iberian Peninsula, where shows its highest intraspecific genetic and phenotypic variation^27–30^. This likely resulted from the complex Iberian physiography and the climatic oscillations during the Pleistocene, which promoted allopatric divergence in populations isolated within Iberian glacial refugia^30,31^. In this region, *S. salamandra* shows a remarkable intraspecific variation in external morphology (e.g. body size, head shape, colour pattern)^28–30^, and more strikingly, in reproductive modes^27^. Two viviparous strategies co-occur^27^: (1) an aquatic-breeding and widespread reproductive mode, larviparity (parturition of aquatic larvae); and (2) a terrestrial-breeding and spatially restricted reproductive mode, pueriparity (parturition of terrestrial juveniles). Pueriparity in *S. salamandra* evolved at least twice: in the subspecies *S. s. bernardezi* along the Cantabrian Mountains during the Pleistocene^31^, and in two Atlantic islands in the subspecies *S. s. gallaica* during the Holocene^32,33^. *Salamandra s. bernardezi* is currently surrounded by larviparous populations of *S. s. gallaica* and *S. s. bejarae* along its western and southern range, respectively. Whether *S. salamandra* populations across the southern slopes of the Cantabrian Mountains belong to *S. s. bejarae* (type locality in the Iberian Central System), or to an undescribed subspecies within the *S. s. gallaica* complex, needs taxonomic revision. However, they both represent independent and closely related lineages within one of the two main *S. salamandra* nuclear clades^30^ and, hence, we opted to consider those populations as traditionally *S. s. bejarae*. The second nuclear clade includes the Iberian *S. s. bernardezi* and *S. s. fastuosa*, together with the Appenninic subspecies *S. s. gigliolii*. On the easternmost range, *S. s. bernardezi* (Asturias-Cantabria border) meets *S. s. fastuosa*, which is considered larviparous despite exhibiting both reproductive strategies in some populations^27, 34^ (Fig. 1). Thus, the northern Iberian populations of fire salamanders constitute an exceptional natural system, in which multiple lineages and phenotypes meet across heterogeneous contact zones in a relatively continuous range, and as such, provide an unusual opportunity to evaluate how exogenous and endogenous factors shape patterns of introgression at the subspecific level.

Here we investigate patterns of gene flow and introgression, and the underlying environmental drivers, between the pueriparous *S. s. bernardezi* and its three larviparous subspecies neighbours across three contact zones in Northern Spain: (1) northern Galicia, where *S. s. bernardezi* is in contact at west of its range with *S. s. gallaica*^35^; (2) the Cantabrian Mountains, where southern populations of *S. s. bernardezi* meet *S. s. bejarae* at relatively high elevations; and (3) the Asturias-Cantabria border where admixture between eastern populations of *S. s. bernardezi* and *S. s. fastuosa* was previously reported^31^. We used a fine-scale individual sampling, and combined one mitochondrial gene (cytochrome b) and 15 microsatellites markers in a landscape genetics approach to: (1) delimit and characterise the range of *S. s. bernardezi*; (2) identify patterns of introgression and quantify levels of admixture between pueriparous and larviparous subspecies in each contact zone; and (3) evaluate the role of environmental and geographic factors in explaining genetic admixture between the different subspecies. Considering previous studies examining phenotypic and/or genetic patterns across northern Iberian populations of *S. salamandra*^29,31,35–38^, we hypothesize that some degree of genetic admixture occurs between subspecies in all contact zones. However, we expect the level of admixture to depend upon the environmental heterogeneity in each area and, therefore, we expect reduced rates of hybridization under IBE or IBR scenarios. Moreover, because of the reduced population connectivity among pueriparous *S. s. bernardezi* populations^38^, we hypothesise that introgression between larviparous and pueriparous populations will be asymmetric.

## Material and methods

### Study system

*Salamandra s. bernardezi* and *S. s. fastuosa* are relatively small salamanders (body size up to 180 mm^27^), with stripe colouration pattern (dorsal yellow and black bands), and round snout^29^. While the former is strictly pueriparous^27,37^, the latter is mostly larviparous despite also presenting pueriparity across its range^34^ (Fig. 1)*.* Conversely, *S. s. gallaica* and *S. s. bejarae* are large salamanders (body size up to 250 mm), show a spotted or blotched colouration pattern and a pointed snout^29^. These two subspecies are larviparous, although insular pueriparous populations of S. *s. gallaica* exist^32,33^.

Despite being phenotypically different in northern Spain, these subspecies inhabit similar habitats, particularly, deciduous woodlands (*Quercus* spp. and *Fagus* spp.) with nearby aquatic systems (streams or ponds) where larviparous females can give birth to aquatic larvae^27,39^. They can also be found in other terrestrial habitats, such as scrublands and coniferous forests, alpine scrublands, and more rarely in eucalyptus (*Eucalyptus* spp.) plantations^39,40^. Previous studies showed agricultural and urban areas comprise strong barriers to dispersal and gene flow in both larviparous and pueriparous fire salamanders^38,41,42^. However, pueriparous salamanders can also survive in harsh environments where water bodies are absent^34,42^, despite water courses (streams and rivers) comprising semi-permeable obstacles to dispersal due to their fully terrestrial life style^38^.

### Study areas and sampling design

**Figure 2:**
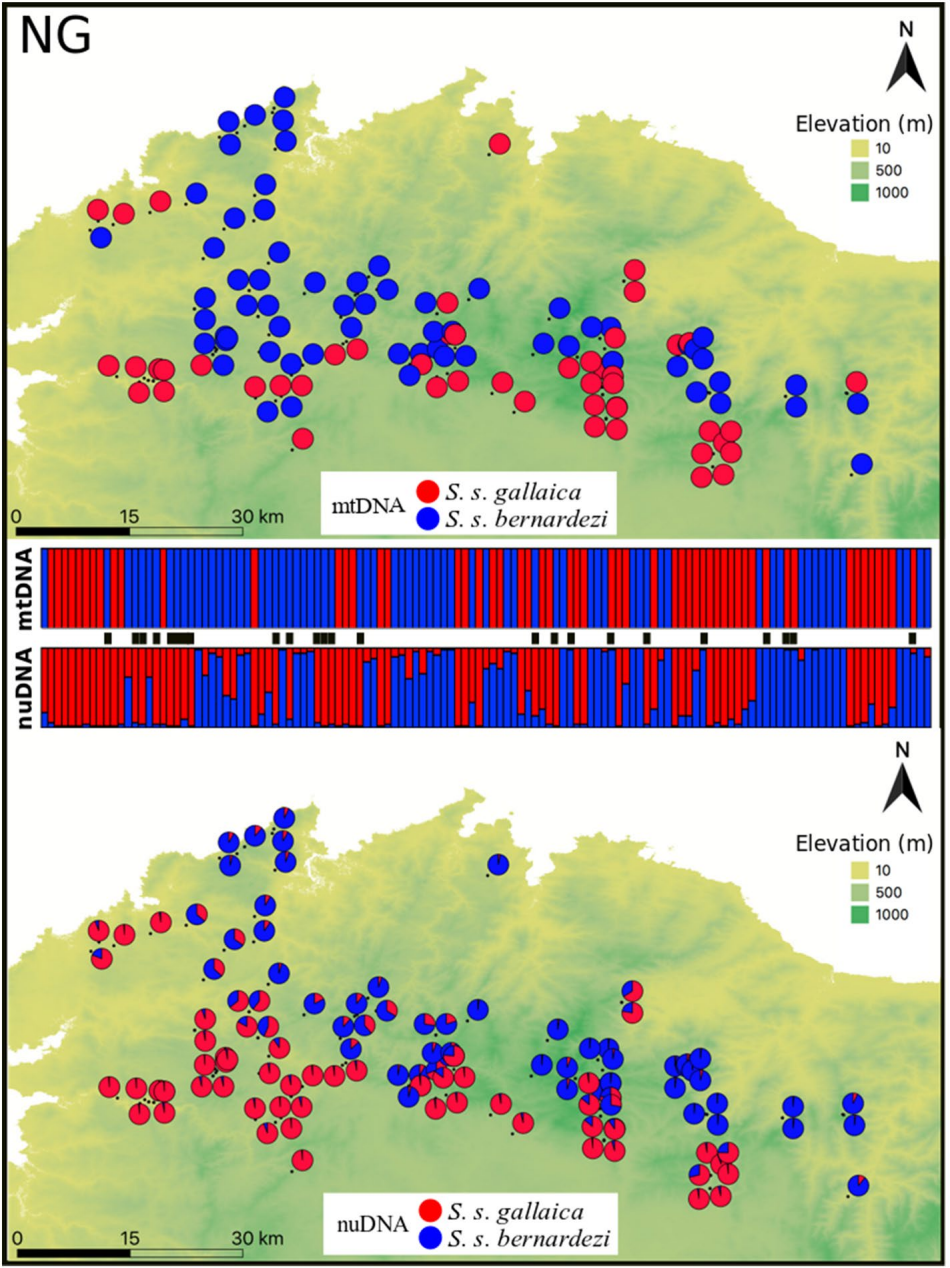
Genetic structure of *S. salamandra* across northern Galician populations (NG) as inferred by both mitochondrial (upper panel; two main lineages separating *S. s. bernardezi* and *S. s. gallaica*) and nuclear DNA (lower panel; STRUCTURE K = 2). Samples in the barplots are arranged from west to east. Genetic assignment to *S. s. bernardezi* or *S. s. gallaica* is colour-coded in blue and red, respectively. Black bars between structure plots indicate individuals with mitonuclear discordance (19%). The figure was created with QGIS software (version 3.2; https://qgis.org/en/site/).

**Figure 3:**
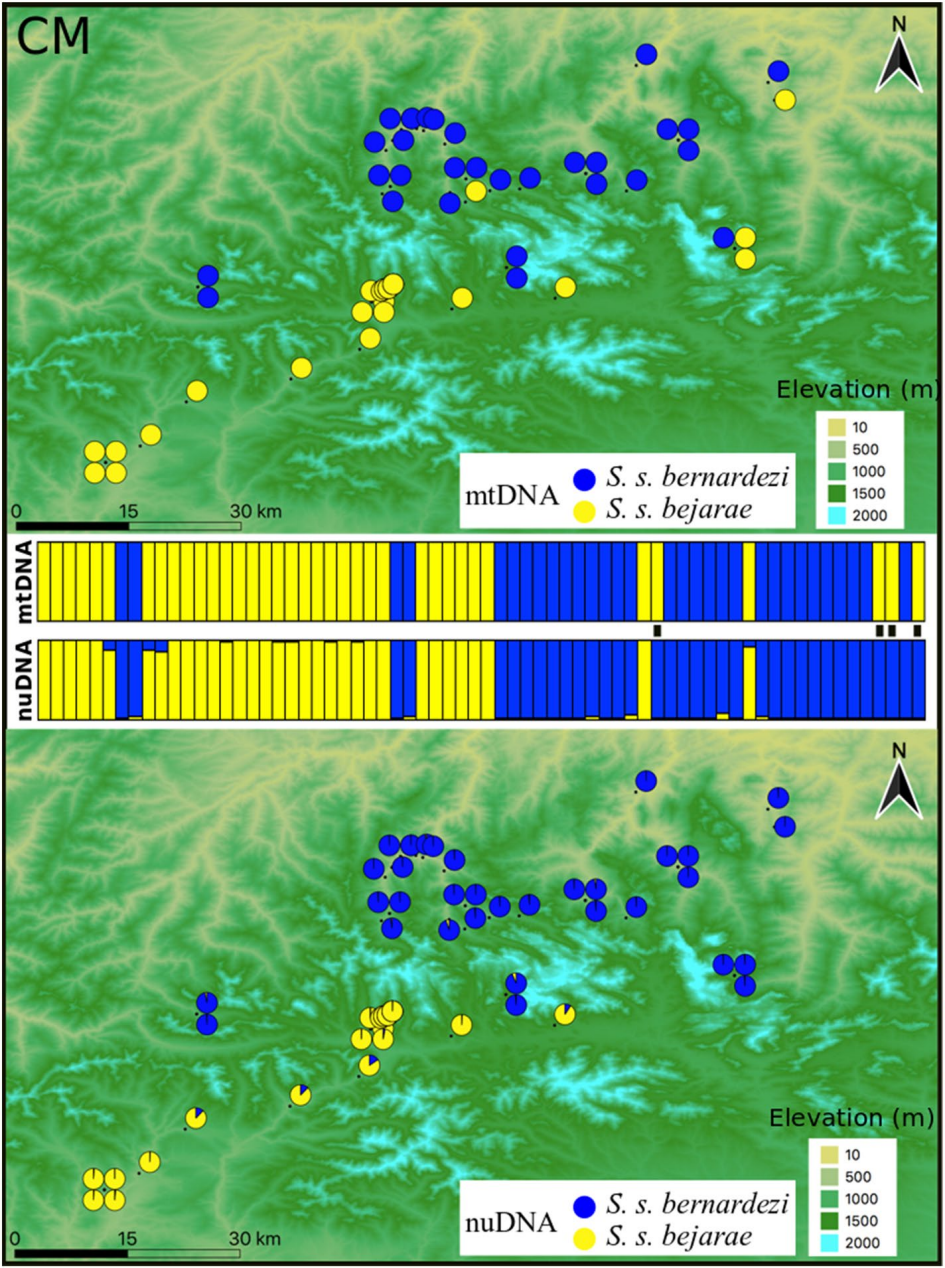
Genetic structure of *S. salamandra* across the Cantabrian Mountains (CM) as inferred by both mitochondrial (upper panel; two main lineages separating *S. s. bernardezi* and *S. s. bejarae*) and nuclear DNA (lower panel; STRUCTURE K = 2). Samples in the barplots are arranged from west to east. Genetic assignment to *S. s. bernardezi* or *S. s. bejarae* is colour-coded in blue and yellow, respectively. Black bars between structure plots indicate individuals with mitonuclear discordance (6%). The figure was created with QGIS software (version 3.2; https://qgis.org/en/site/).

**Figure 4:**
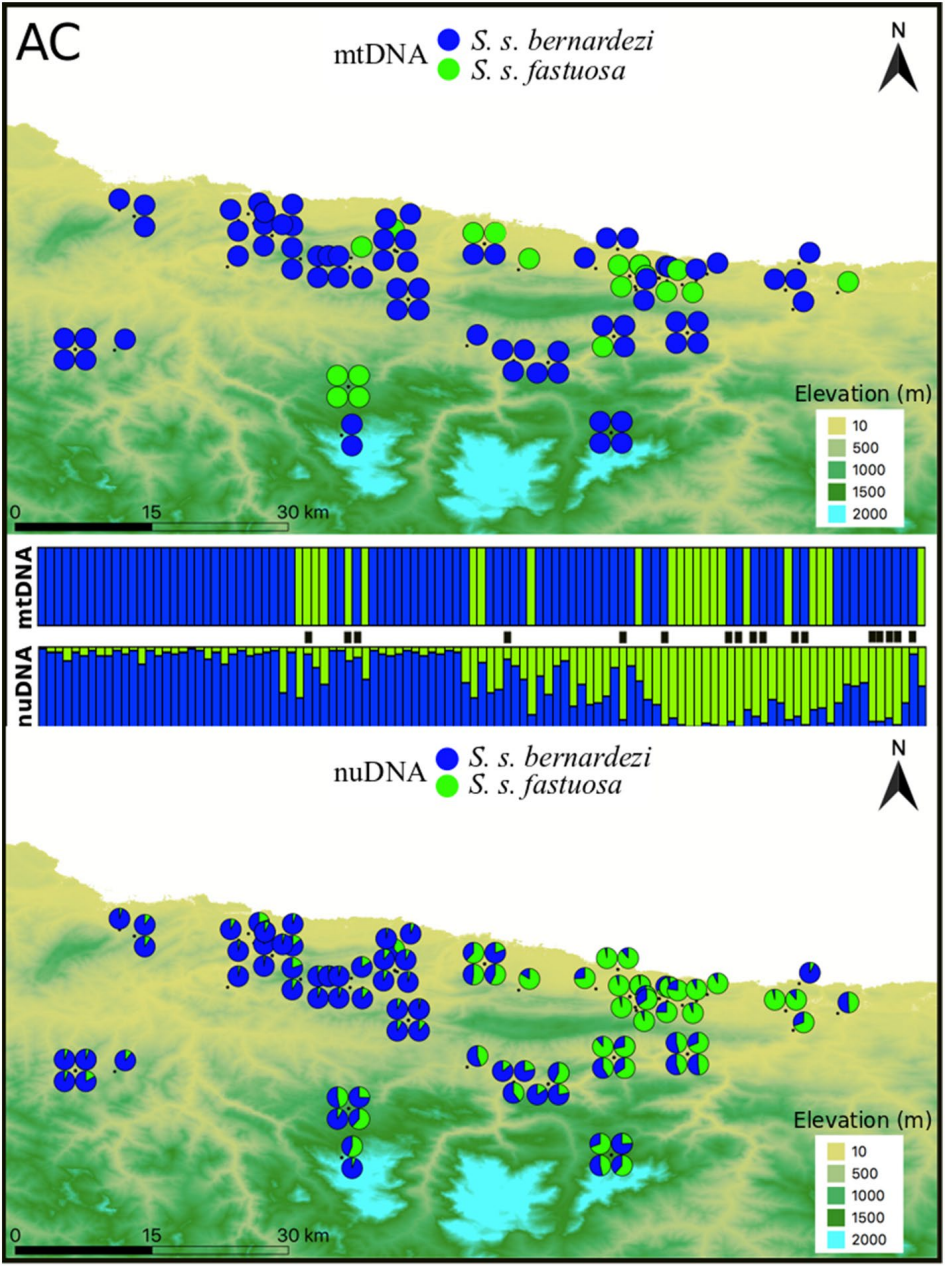
Genetic structure of *S. salamandra* across the Asturian-Cantabrian border (AC) as inferred by both mitochondrial (upper panel; two main lineages separating *S. s. bernardezi* and *S. s. fastuosa*) and nuclear DNA (lower panel; STRUCTURE K = 2). Samples in the barplots are arranged from west to east. Genetic assignment to *S. s. bernardezi* and *S. s. fastuosa* is colour-coded in blue and green, respectively. Black bars between structure plots indicate individuals with mitonuclear discordance (16%). The figure was created with QGIS software (version 3.2; https://qgis.org/en/site/).

We carried out individual-based sampling (1–3 samples per locality) within each contact zone because this sampling approach was shown to provide greater statistical power in detecting landscape genetic patterns compared to population-based sampling^43^. We selected three heterogeneous study areas (i.e. different levels of environmental and habitat variability) where different subspecies, morphotypes and the two distinct reproductive modes meet, thus covering a large portion of the secondary contact zones between *S. s. bernardezi* and its neighbouring lineages (Fig. 1). We performed sampling during rainy nights from November 2013 until May 2015 (Supplementary Table S1). We sampled mostly adult individuals (toe clip), but on a few occasions we also sampled larvae (tail clip). Tissue samples were stored in absolute ethanol and all individuals were released in their sampling points. At the western range of *S. s. bernardezi*, we collected samples of 129 salamanders from 86 localities following a west–east axis along northern Galicia (NG hereafter), where intermixed phenotypes between *S. s. bernardezi* and *S. s. gallaica* were previously observed^32,35^ (Fig. 2). The landscape in NG is relatively flat (elevation ranges from 0 m up to 1016 m a.s.l. using the minimum extent of the samples, Supplementary Fig. S1) and is extensively fragmented by agricultural practices and *Eucalyptus* spp. plantations. Remnant populations of fire salamanders in NG occur in suitable forested and scrubland patches in lowland and hilly areas. At the southern range of *S. s. bernardezi*, we also followed a longitudinal sampling along the Cantabrian Mountain range (CM hereafter), where we collected samples of 73 individuals from 38 localities on both sides of the mountains, representing *S. s. bernardezi* and *S. s. bejarae* from the northern and southern slopes, respectively (Fig. 3). The landscape in this study plot is characterized by high elevation, steep terrains, and high topographic complexity (elevation ranging from 179 m up to 2343 m above sea level (a.s.l.), Supplementary Fig. S1), and is highly suitable for the species due to the native forests and scrublands that dominate the region with sparse agricultural patches. At the eastern range of *S. s. bernardezi*, we obtained samples of 123 individuals from 58 localities across the Asturias-Cantabria border (AC hereafter, Fig. 4). We did not extend the sampling further south to avoid sampling *S. s. bejarae* (Fig. 1). Despite the high elevation in this region (elevation ranging from 0 m up to 2394 m a.s.l., Supplementary Fig. S1), the mountains follow a west–east direction and thus, no major topographic barriers exist between these two subspecies, which frequently occur along coastal valleys. The AC area is composed of natural forests and scrublands, with eucalypt plantations, agricultural areas and human infrastructures (e.g. villages, roads) being predominant on the coast.

Fieldwork for obtaining tissue samples was done with the corresponding permits from the regional administrations (Xunta de Galicia, Ref. 024/2014, and 410/2015; Gobierno del Principado de Asturias, Ref. 2018/2115; Castilla y León, EP/CYL/625/2013, EP/CYL/725/2015; Picos de Europa National Park CO/09/0125/2013, CO/09/012/2014, CO/09/065/2015). Sampling procedures were carried out following the Guidelines for Use of Live Amphibians and Reptiles in Field and Laboratory Research, 2nd Edition, revised by the Herpetological Animal Care and Use Committee (HACC) of the American Society of Ichthyologists and Herpetologists, 2004. All procedures complied with the country legal requirements on animal welfare (RD 53/2013) and were conducted in accordance with the guidelines of the Research Ethics Committee of the University of Oviedo (Comité Ético de Experimentación Animal de la Universidad de Oviedo) under authorisation #8-INV-2012. The members of the research team have approved licenses by the Service of Animal Welfare and Production of the Principality of Asturias to conduct experimental protocols with animals (license types C and D to A.G.N). This study was carried out in compliance with the ARRIVE guidelines (Animal Research: Reporting in Vivo Experiments) for how to report animal research in scientific publications (https://arriveguidelines.org/arrive-guidelines).

### Molecular markers and laboratory procedures

We extracted genomic DNA using the Genomic DNA Tissue Kit (EasySpin), following the manufacturer’s protocol, and verified both the quantity and quality of extracted products in a 0.8% agarose gel. We used the DNA extract as template for polymerase chain reactions (PCR) to amplify and sequence one mitochondrial (mtDNA) fragment (ca. 1100 bp) of the cytochrome b and adjacent tRNAs (hereafter cytb). We used the primers Glu14100L and Pro15500H^44^ to amplify cytb following a previously described protocol^28^. We outsourced DNA sequencing to Genewiz Inc. (Leipzig, Germany), and inspected and aligned resulting chromatograms using GENEIOUS version 11.1.4 (http://www.geneious.com). Extracted products were also used to amplify and genotype 15 microsatellite loci (SST-A6-I, SST-A6-II, SST-B11, SST-C3, and SST-G9^45^; SalE14, Sal29, SalE12, SalE7, SalE5, SalE2, SalE06, Sal3, and SalE8^46^) distributed in five optimized multiplexes (see^47^ for details about the protocol optimisation and genotyping).

Our individual-based approach, together with some degree of continuous phenotypic variation in the contact zones, prevent us from grouping samples into a priori populations or genetic units, and thus from assessing accurately Hardy–Weinberg equilibrium (HWE) and linkage equilibrium (LE). However, previous phylogeographic and population genetic studies using the same set of markers^33,38,41,42,48–50^ on Iberian populations of *S. salamandra* support the validity of these markers for population and landscape genetic analyses.

### Phylogenetic lineages

We used the cytb sequences to perform coalescent-based Bayesian analyses in BEAST version 1.8.4^51^ and identify the mitochondrial lineage (mtDNA) of each sample, which was then used to estimate the extent of cyto-nuclear discordance across each contact zone. However, we note that mtDNA phylogenetic tree does not represent the true phylogenetic relationships among *S. salamandra* subspecies. In particular, *S. s. fastuosa* (with embedded *S. s. gigliolii*) was placed as the sister lineage to *S. s. bernardezi* and formed a well differentiated clade within *S. salamandra* according to phylogenomic data^30^. Yet, both *S. s. gallaica* and *S. s. bejarae* fall within the second clade and are sister lineages in both mtDNA and phylogenomic trees. We selected the optimal nucleotide substitution model (TrN) with JMODELTEST version 2.1.4^52^, under the Bayesian information criterion (BIC). To estimate the time to the most recent common ancestor (TMRCA) of mtDNA lineages we used both a prior substitution rate and fossil calibrations. We set the divergence rate for cytb estimated in a 2.7% Myr for Mediterranean amphibians^53^. We used a normal distribution with a mean of 0.0134 and SD of 0.0009. We also used two calibration points (divergence date estimates) based on the split between *Chioglossa* and *Mertensiella* (mean: 19.4; lower and upper 95% confidence intervals (CIs) of 14.2 and 27.5, respectively), and the split between *Lyciasalamandra* and *Salamandra* (mean: 17.59; 95% CIs 11.24 and 24.82). These estimates were obtained from a two-step protocol based on a combination of primary fossil calibrations and secondary calibrations from a multilocus nuclear tree on a multilocus mitochondrial tree^53^. However, we avoided the use of internal nodes provided within *Salamandra* due to some incorrect phylogenetic relationships at both inter-specific (e.g. *S. algira*) and intra-subspecific (e.g. *S. s. morenica*) levels^30^. We obtained cytb sequences from GenBank for *Chioglossa lustitanica* (AF329314), *Mertensiella caucasica* (EU880319), *Lyciasalamandra flavimembris* (EU880318) and *Lyciasalamandra atifi* (AF154053), which were added to our cytb alignment. We performed three independent runs using an uncorrelated relaxed clock and a constant population size model as the coalescent tree prior, with a total of 100 million generations (burn-in: 10%). We combined tree files of all runs using the software LOGCOMBINER version 1.7.5, and verified parameter convergence by examining the effective sample sizes (ESSs) in TRACER version 1.6. We obtained a maximum clade credibility summary tree with Bayesian posterior probabilities (BPP) for each node using TREEANNOTATOR version 1.8.4, and edited the resulted tree in FIGTREE version 1.4.3 (http://tree.bio.ed.ac.uk/software/figtree). We ran BEAST analyses in the CIPRES Science Gateway^54^. We estimated genetic distances (uncorrected p-distances) between subspecies in GENEIOUS 11.1.4 (www.geneious.com).

### Genetic structure and hybridization analysis

We first used STRUCTURE 2.3.4^55^ to assess the individual genetic membership coefficient (q-value) for each subspecies in each contact zone. Because we were not interested in determining the genetic structure of each study area but rather assigning individuals to one of the two co-occurring subspecies, we ran STRUCTURE for K = 2 (two clusters). We ran 10 replicates and used the correlated allele frequency and admixture models, without location prior, and with a burn-in period of 100,000 iterations followed by 1,000,000 iterations. We combined the results from the 10 replicate runs using CLUMPP^56^. To complement STRUCTURE, we used the spatially explicit method of spatial Principal Component Analyses (sPCA)^57^, which does not assume HWE or LE expectations. To identify if the sPCA scores were able to assess global (patches and clines where neighbours tend to be similar) and local (strong genetic differences between neighbours) structure, we used a Delaunay triangulation connection network and two separate Monte Carlo tests with 100 permutations.

Because each individual has two q-values (one for each of K=2 clusters), we considered individuals as admixed when showing low levels of coefficient membership for either of the two clusters (0.1 < q-value < 0.9). Then, we used these potential hybrids to estimate the probability of assignment (Z) of early-generation hybrid or backcrossed individuals between subspecies within each contact zone in NEWHYBRIDS v.1.1^58,59^. We used six classes in each analysis: pure individuals of each subspecies (parental classes), first generation hybrid (F1), second-generation hybrid (F2), and first-generation backcrosses to each subspecies. To increase the power to detect hybrids, we also assigned all individuals with > 90% (q-value > 0.9) estimated ancestry in STRUCTURE from a single subspecies to the appropriate pure parental category using the ‘z’ option. We performed three independent runs per contact zone and we set 1 million generations with a burn-in of 10% per run. We used two random starting values in each NEWHYBRIDS run, and the “Jeffreys-like” prior for the estimates of θ (i.e., allele frequencies) and π (i.e., mixing proportions). We used a posterior probability threshold of P ≥ 0.7^60^ to assign potential hybrids identified in STRUCTURE to each of the six classes.

### Landscape genetics analysis

**Figure 5:**
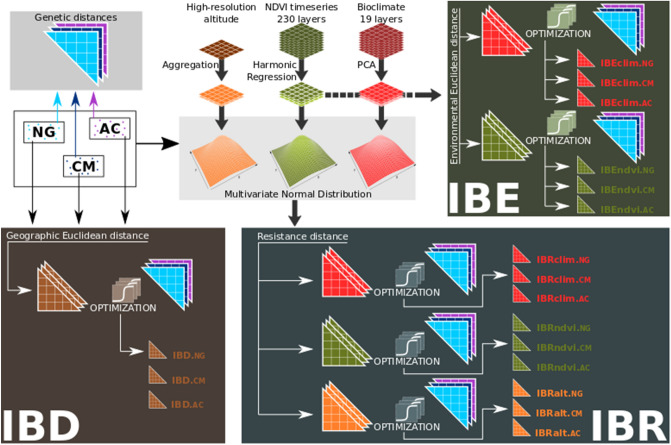
Landscape genetics analyses workflow used to derive optimized variables representing the different isolation models (IBD, IBR, and IBE). Two parameters of a logistic curve (curvature and inflection point) were applied to the isolation by distance matrix and optimized with genetic algorithms. The objective of optimization is the maximization of the logLikelihood from a GLS regression. Note that this workflow is for a single genetic distance type, thus, it was executed twice for the R_QG_ and R_TRI_ genetic distances. The figure was created with Inkscape software (version 1.0.2; https://inkscape.org/).

The workflow for the landscape genetic analyses is summarised in Fig. 5 and detailed as follows.

#### Genetic distance matrices

The use of individual-based distances is recommended to avoid potential violations of the assumption of discrete populations that can lead to erroneous estimates of genetic parameters^61^. We used COANCESTRY^62^ to calculate pairwise relatedness matrices between individuals in each studied contact zone. First, we used the relatedness coefficient from Queller & Goodnight (1989) (R_QG_)^63^, which does not assume Hardy–Weinberg equilibrium. We also used the triadic likelihood relatedness (R_TRI_) estimate^64^, which calculates pairwise relatedness between two individuals while using a third one as control, thus reducing the identical by descent error rate. The matrices were linearly scaled to the range between 0 and 1, where higher values indicate higher similarity. To obtain an indication of dissimilarity, we found the complement of each matrix element by subtracting to 1.

#### Spatial data

We evaluated the three main environmental features that can influence spatial connectivity and gene flow of fire salamanders across the study region: elevation, habitat and climate. Elevation data was obtained from the Spanish *Instituto Geográfico Nacional* (https://www.ign.es) at a 5 m resolution and aggregated to a 250 m resolution for downstream analyses. We produced two variables using a different aggregating function: (1) the average to represent mean elevation at each 250 m pixel, and (2) the standard deviation as a representation of the topographic heterogeneity. This collection of two variables is hereafter referred to as elevation data. We used the Normalized Difference Vegetation Index (NDVI) as a continuous surrogate of habitat availability. The NDVI imagery was obtained with a 16-day period covering a range of 10 years (from January 2009 to December 2018) that  includes the field work period. The MOD13Q1 product was downloaded from the EarthData website (https://earthdata.nasa.gov/) with a spatial resolution of 250 m and 16-day temporal resolution, resulting in a time-series with 230 measurement layers. To provide summaries of the 16-day NDVI for a few variables, we processed the data with a harmonic regression and extracted three spatial coefficients representing the periodic behaviour in each pixel as continuous surrogates of local habitat^65^. The collection of three variables is hereafter referred as NDVI data. All data was processed in R with packages *rgdal* (v.1.5.8), *raster* (v.3.3.13), *TSA* (v.1.2.1) and *zoo* (v.1.8.8). Climate data was obtained from the CHELSA database (https://chelsa-climate.org/^66^) as 19 bioclimate variables at 30” (~ 1 km) spatial resolution. We further summarized the climate data by means of a PCA after centering and scaling. Resulting spatial components from PCA are hereafter referred to as the climate data. We projected all spatial data to the WGS84 coordinate system.

#### Isolation by distance, environment and resistance matrices

We generated geographic, environmental and resistance distance matrices to test for the dominant model of isolation by distance (IBD), environment (IBE), or resistance (IBR) in each contact zone. We produced IBD matrices by calculating Euclidean geographic distances between genotyped individuals in each contact zone. We transformed coordinates to the ETRS89 Lambert Azimuthal Equal-Area projection before calculating the straight line distance between sampling localities.

To account for environmental differentiation between sampling locations, we calculated one IBE matrix based on climate (IBE_CLIM_) and another based on NDVI (IBE_NDVI_) for each contact zone (six IBE matrices in total; see Fig. 5). The IBE matrices were calculated as the Euclidean distance in the environmental space^6,67^. As such, we extracted at the locations of samples the value of the PCA components from climate data and calculated the distances between them. We followed the same procedure with NDVI data.

We generated IBR matrices in relation to descriptive categories: elevation (IBR_ALT_), climate (IBR_CLIM_), and NDVI (IBR_NDVI_). We used a common dataset combining the presence locations from the three contact zones to determine the usage of each variable in each category. Pixel duplicates were removed independently in each category due to the different spatial resolution. We extracted variable data at the presence locations and calculated a vector of average values per variable and the covariance matrix. We used this information to combine the different variables by means of a multivariate normal distribution, generating a map representing the likelihood of finding a presence for each variable category. Hence, this value is related to the usage of the multidimensional space of each combination of variables by the species. We used these maps as a resistance surfaces, after a minmax scaling, in order to generate resistance distances with the ‘*commuteDistance*’ function in ‘*gdistance*’ R package (v.1.3.6).

#### Optimizations and model selection

Parameter optimization procedures are common in landscape genetic studies^68–71^. These optimizations aim at finding find the best combination of parameters describing a species’ environmental preferences to a resistance surface that has the best fit with genetic data. Often the aims of the optimization procedure are the parameters of a linear combination of habitat and other niche descriptors by maximizing a value relating the derived resistance distances to the genetic distances^68,70^. Optimizations algorithms have also been used to find the best parameters of a transformation function applied to the resistance distances^69,71^. Usually only the matrix referring to IBR is optimized, hence, it has an advantage over other competing models under a test of the isolation mechanism that better explains genetic divergence. Here we optimized the parameters of a logistic function relating each matrix produced (IBD, IBE and IBR) to the genetic distance matrices in each contact zone. It is expected that genetic differentiation increases with geographical distance. Yet, due to limited movement and finite population sizes and genomes, this increase cannot occur indefinitely and tends to plateau^72^. The same reasoning can be applied to the other models, including resistance and environmental distances. We optimized a logistic function with two parameters (curvature and inflection point), allowing for a near linear arrangement with original distances to a full logistic relation with steep transition. In the latter case, larger distances tend to plateau after the inflection point thus allowing a scenario where genetic differentiation accumulates until a maximum limit. We performed optimizations with genetic algorithms using the R package *GA* (v.3.2) to maximize the logLikelihood of a Generalized Least Squares (GLS) regression using the genetic matrix as a dependent variable and an isolation matrix as an independent variable. Data in pairwise distance matrices show high degrees of non-independence which hampers the direct application of regression-based algorithms. The GLS regression incorporates correlation structures informing about the lack of independence between samples. We used a maximum-likelihood population effects (MLPE) model that generates such correlation structure from pairwise data^71^. We used the package *nlme* (v.3.1.140) for GLS with *corMLPE* (v.0.0.3, https://github.com/nspope/corMLPE). All optimizations were done with a maximum of 1000 iterations or 200 runs without change of the optimization score. All isolation model matrices were min–max scaled to the range 0–1 in order to facilitate the comparison of the optimized parameters.

We used the Akaike Information Criterion (AIC) to rank univariate models and to eliminate those isolation models that were contributing less to the overall fit of the regression (Table 2). Models with ΔAIC lower or equal to 2 were assumed to be equivalent to best model. By comparing only univariate models and the null model, we do not use competing scenarios of resistance in the same model.

## Results

### Phylogenetic analysis

We obtained a total of 302 sequences after discarding sequences of poor quality and trimming some sequences (from ca. 1070 bp to 350 bp). Phylogenetic analyses identified two main monophyletic (Posterior Probability > 0.95) clusters within the three studied contact zones: one includes seven well supported mtDNA lineages of putative pueriparous *S. s. bernardezi*, while the second clade includes three monophyletic lineages of the three focal larviparous subspecies (*S. s. gallaica*, *S. s. bejarae* and *S. s. fastuosa*) (Fig. 1). According to the cytb dating approach, the split between *S. s. bernardezi* and the monophyletic clade including *S. s. gallaica*, *S. s. bejarae* and *S. s. fastuosa* is estimated at 1.3 Mya (0.9–1.7) with an earlier diversification within *S. s. bernardezi* (ca. 1 Mya [0.8–1.4]) compared to the *S. s. gallaica*-*bejarae*-*fastuosa* clade (ca. 0.3 Mya [0.15–0.4]).

### Nuclear genetic structure, cyto-nuclear discordances and hybridization

After discarding individuals with missing data > 10% in the microsatellite dataset, we obtained a fully comparative mtDNA-nuDNA datasets for a total of 302 samples (127, 68 and 107 from NG, CM and AC, respectively).

STRUCTURE recovered two well defined genetic groups in each contact zone that largely match the distribution of each mitochondrial lineage (Figs. 2, 3, 4). The first eigenvalue of sPCA analyses confirmed the genetic structure found in STRUCTURE, which divided the data into two groups for each contact zone (Supplementary Figs. S2–S4). We found a significant global structure in each dataset but not significant support for a local structure in sPCA.

We found relatively similar patterns of hybridization in the contact zones of NG and AC (Table 1), in which, relatively high proportions of admixed individuals and cyto-nuclear discordances were inferred. Specifically, for NG, from the 127 analysed salamanders, a total of 92 individuals (72%) were assigned either to the subspecies *S. s. gallaica* (49 salamanders; 38%) or *S. s. bernardezi* (43 salamanders; 34%), while the remaining 35 salamanders (28%) were inferred as potential hybrids (0.1 ≤ q-value ≤ 0.9 in STRUCTURE). Among these potential hybrids, 29 salamanders were labelled as F2 hybrids in NEWHYBRIDS. Similarly, in AC, from the 107 genotypes, 57 salamanders (53%) were assigned either to the subspecies *S. s. fastuosa* (16 individuals; 15%) or *S. s. bernardezi* (41 salamanders; 38%). The remaining 50 salamanders comprised potential hybrids (47%), from which, 38 individuals were categorised as F2 hybrids, two salamanders as pure *S. s. bernardezi*, and two others as *S. s. fastuosa* in NEWHYBRIDS. Unlike the latter two contact zones, CM was characterized by a very low proportion of admixed individuals. From the 68 studied salamanders, a total of 65 individuals (96%) were assigned either to the subspecies *S. s. bejarae* (29 individuals; 43%) or *S. s. bernardezi* (36 salamanders; 53%), while the remaining three salamanders (4%) that were inferred as potential hybrids were later assigned as pure *S. s. bejarae* in NEWHYBRIDS. Furthermore, the number of cyto-nuclear discordances was higher for NG and AC (19% and 16%, respectively) than for CM (6%).

**Table 1:** Genetic assignments based on STRUCTURE (Pure 1, Pure 2 and Hybrids; with both total numbers and percentages by class), and NEWHYBRIDS (F1, F2, backcrosses; total numbers by class).

Contact Zone	STRUCTURE			NEWHYBRIDS			
	Pure 1	Pure 2	Hybrid	Hybrid class			
NG	*S. s. bernardezi*	*S. s. gallaica*	*bernardezi/gallaica*	F1	F2	BC-*bernardezi*	BC-*gallaica*
	43 (34%)	49 (38%)	35 (28%)	0	29	0	0
CM	*S. s. bernardezi*	*S. s. bejarae*	*bernardezi/bejarae*	F1	F2	BC-*bernardezi*	BC-*bejarae*
	36 (53%)	29 (43%)	3 (4%)	0	0	0	0
**AC**	*S. s. bernardezi*	*S. s. fastuosa*	*bernardezi/fastuosa*	F1	F2	BC-*bernardezi*	BC-*fastuosa*
	41 (38%)	16 (15%)	50 (47%)	0	38	0	0

### Landscape genetics analyses

The PCA of climate data showed the first three components accounted for 91.4% of total variation. Optimizations of IBD and IBE models showed a high degree of success, with the ΔAIC of the optimized model showing values higher than 15 compared to the non-optimized version (see Supplementary Table S2). The IBR model optimization showed a differential success depending on the contact zone. In the contact zone NG, all IBR models achieved a ΔAIC lower than 4.5, revealing a lack of effect of the optimization. A single IBD univariate model was selected as the best model based on ΔAIC values for the contact zones of NG and AC, independently of the genetic matrix used (Table 2). The contact zone of CM comprises a more complex scenario because univariate models indicate a dominant action of IBR in addition to IBD, particularly IBR_CLIM_ and IBR_NDVI_ for R_QG_ genetic distances, and IBR_CLIM_ with IBD for R_TRI_ (Table 2). The combinations of the best models suggest not only a strong effect of IBD in all contact zones, but also a strong effect of habitat and climate resistance across the CM contact zone. The relationship between pairwise genetic distance (for both R_QG_ and R_TRI_) and pairwise geographic and environmental distances for each contact zone are represented in Supplementary Figs. S5 and S6.

**Table 2:** Results of the GLS regression with MLPE correlation relating the genetic distance with different models of isolation (IBD, IBE and IBR). Predictors showing at least one best model are highlighted in bold.

CZ	Predictors	df	logL		AIC		∆AIC		AIC weights		logL ratio		*p* value	
			*QG*	*TRI*	*QG*	*TRI*	*QG*	*TRI*	*QG*	*TRI*	*QG*	*TRI*	*QG*	*TRI*
NG	intercept	3	− 11,211.27	− 11,323.50	22,428.54	22,652.99	952.58	712.32	0.00	0.00	0.00	0.00	1.00	1.00
	**IBD**	4	− 10,733.98	− 10,966.34	21,475.96	21,940.67	**0.00**	**0.00**	1.00	1.00	954.58	714.32	0.00	0.00
	IBE_CLIM_	4	− 11,036.76	− 11,169.68	22,081.52	22,347.36	605.56	406.69	0.00	0.00	349.02	307.63	0.00	0.00
	IBE_NDVI_	4	− 11,165.25	− 11,271.59	22,338.50	22,551.18	862.54	610.51	0.00	0.00	92.04	103.81	0.00	0.00
	IBR_CLIM_	4	− 10,869.67	− 11,081.17	21,747.35	22,170.35	271.39	229.68	0.00	0.00	683.19	484.65	0.00	0.00
	IBR_NDVI_	4	− 11,211.09	− 11,323.06	22,430.18	22,654.13	954.22	713.46	0.00	0.00	0.36	0.86	0.55	0.35
	IBR_ALT_	4	− 10,807.98	− 11,039.25	21,623.96	22,086.50	148.00	145.83	0.00	0.00	806.58	568.49	0.00	0.00
CM	intercept	3	− 3205.62	− 3219.53	6417.24	6445.06	621.94	430.11	0.00	0.00	0.00	0.00	1.00	1.00
	**IBD**	4	− 2901.41	− 3003.48	5810.81	6014.95	15.51	**0.00**	0.00	0.72	608.43	432.11	0.00	0.00
	IBE_CLIM_	4	− 3034.30	− 3102.08	6076.60	6212.16	281.30	197.20	0.00	0.00	342.64	234.91	0.00	0.00
	IBE_NDVI_	4	− 3012.16	− 3078.95	6032.33	6165.90	237.02	150.94	0.00	0.00	386.92	281.17	0.00	0.00
	**IBR**_**CLIM**_	4	− 2894.05	− 3004.44	5796.09	6016.87	**0.79**	**1.92**	0.40	0.28	623.15	430.19	0.00	0.00
	**IBR**_**NDVI**_	4	− 2893.65	− 3066.47	5795.31	6140.94	**0.00**	125.99	0.60	0.00	623.94	306.12	0.00	0.00
	IBR_ALT_	4	− 2948.22	− 3057.46	5904.43	6122.91	109.13	107.96	0.00	0.00	514.81	324.15	0.00	0.00
AC	intercept	3	− 7948.01	− 8024.00	15,902.02	16,054.00	1084.70	831.60	0.00	0.00	0.00	0.00	1.00	1.00
	**IBD**	4	− 7404.66	− 7607.20	14,817.32	15,222.40	**0.00**	**0.00**	1.00	1.00	1086.70	833.60	0.00	0.00
	IBE_CLIM_	4	− 7853.25	− 7930.42	15,714.51	15,868.84	897.19	646.44	0.00	0.00	189.51	187.16	0.00	0.00
	IBE_NDVI_	4	− 7882.72	− 7957.05	15,773.44	15,922.10	956.13	699.70	0.00	0.00	130.57	133.90	0.00	0.00
	IBR_CLIM_	4	− 7913.07	− 8013.11	15,834.14	16,034.22	1016.82	811.82	0.00	0.00	69.88	21.78	0.00	0.00
	IBR_NDVI_	4	− 7945.03	− 8022.24	15,898.07	16,052.47	1080.75	830.07	0.00	0.00	5.95	3.53	0.02	0.06
	IBR_ALT_	4	− 7429.34	− 7676.98	14,866.68	15,361.96	49.36	139.56	0.00	0.00	1037.34	694.04	0.00	0.00

The log Likelihood (logL) ratio compares each model with an intercept-only model. The AIC differences (∆AIC) is in respect to the best model (lowest AIC) in each combination of genetic distance matrix and contact zone. Models with ΔAIC lower or equal to 2 (best model) are highlighted in bold.

## Discussion

The degree of reproductive isolation is expected to be larger between lineages showing differentiation in parity modes^73^, morphology^74^ or behaviour^75^, though time since divergence also enhances reproductive barriers^76^. Our fine-scale sampling and molecular approach contributes to an accurate characterisation of the location and spatial extent of the three studied contact zones, where the morphologically^29^ and phylogenetically^30^ divergent pueriparous *S. s. bernardezi* meets three larviparous subspecies (*S. s. gallaica*, *S. s. bejarae*, and *S. s. fastuosa*). We detected clear signs of gene flow and introgression in two out of three regions, thus supporting the hypothesis of incomplete reproductive isolation between these highly divergent *S. salamandra* morphotypes. Despite the extensive patterns of introgression and admixture in two contact zones (NG and AC), the Cantabrian Mountains (CM) likely reduce gene flow between the larviparous *S. s. bejarae* and pueriparous *S. s. bernardezi*. This study suggests the combined effect of landscape and climate barriers shape patterns of hybridization, thus highlighting the need of genetic studies across multiple and heterogeneous contact zones to better delineate the spatial patterns of gene flow and hibridisation between non-reproductively isolated taxa.

### Geographic range and spatial extent of *Salamandra salamandra* contact zones

Previous phylogeographic studies suggested the eastern Galician mountains comprise a barrier to gene flow between *S. s. gallaica* and *S. s. bernardezi*^31,32^. The high proportion of admixed individuals inferred from our genetic analyses demonstrates the existence of a contact zone between western *S. s. bernardezi* and northern *S. s. gallaica* populations within a relatively homogenous landscape in northern Galicia (NG), thus indicating previous works were likely biased due to an insufficient sampling in this region. We detected a north–south contact zone between *S. s. bernardezi* and *S. s. gallaica* located ca. 10–30 kms from the northern Galician coast (Fig. 2). The sampled salamanders in NG were largely characterised by a mixture of phenotypes typical of *S. s. gallaica* and *S. s. bernardezi*, as previously documented by morphological studies in this region^35,77^. The coloration pattern across this area varied considerable. Most admixed individuals showed a disrupted striped colouration, resembling *S. s. bernardezi* phenotype, but also showed intermediate colouration patterns with both stripes and blotches. These admixed individuals also showed intermediate body sizes, and variable snout shapes, thus hindering subspecies assignment and supporting the existence of a hybrid zone. Additionally, the presence of females with a mixed reproductive mode (i.e. females give birth to both metamorphosed juveniles and aquatic larvae^35^; unpublished data) corroborates the presence of a hybrid zone in NG.

Patterns of genetic structure confirmed a negligible hybrid zone between southern *S. s. bernardezi* and northern *S. s. bejarae* populations across the Cantabrian Mountains (CM; Fig. 3). Contrary to NG, the contact zone in CM shows an abrupt change of phenotypes between populations inhabiting southern slopes, where fire salamanders are larviparous, large, blotched, with a relatively pointed snout, and populations located on northern slopes, in which individuals exhibit pueriparity, are small, striped and often present rounded snouts^29,31,77^.

Similar to NG, we also found a high proportion of admixed individuals along the Asturias-Cantabria border (AC), where eastern populations of *S. s. bernardezi* hybridize with *S. s. fastuosa* along lowlands (west–east corridors) located between mountain chains (Sierra de Cuera and Picos de Europa; Fig. 4). This area coincides with a secondary contact between amphibian lineages with Atlantic climate affinity (*Lissotriton helveticus*^78^; *Rana parvipalmata*-*R. temporaria*^79^). The deep divergence found in *Rana* points to an old split (4 Mya) and a narrow hybrid zone (25 km), which led the authors to conclude these two lineages represent distinct species. *Salamandra*, however, shows a more recent split (late Pleistocene). Despite this study showing the existence of a contact zone between these subspecies, previous phylogeograhic work placed this hybrid zone farther east to the Pyrenees^30,31^. The identification of the contact zone between *S. s. bernardezi* and *S. s. fastuosa* is not possible at the morphological level because *S. s. fastuosa* appears to be phenotypically indistinguishable from *S. s. bernardezi*. We have also detected females with mixed reproductive modes across AC, and also cases of larviparity and pueriparity within the *S. s. bernardezi* and *S. s. fastuosa* ranges, respectively, which again supports the presence of a relatively broad hybrid zone in this area. Nevertheless, future studies should be conducted to accurately delimit the range of *S. s. fastuosa* and the level of introgression across its range.

### Genetic introgression and gene flow between *S. salamandra* subspecies

Patterns of cyto-nuclear discordances are common in animals^80^, including salamanders. Conflicting geographic patterns between mitochondrial and nuclear genetic markers have been reported across the *S. salamandra* range (i.e. *S. s. bernardezi—S. s. fastuosa*^31^; *S. s. bernardezi—S. s. gallaica*^38^; *S. s. bejarae—S. s. almanzoris*^48^; *S. s. gigliolli—S. s. terrestris*^81^, and between subspecies in its sister species, *S. algira*^82^. Similarly, we found cyto-nuclear discordances across the three studied contact zones, though these were higher in NG and AC than in CM.

Despite the marked genetic^30^ and phenotypic (i.e., colour patterns^35^, morphology^29^, and reproductive modes^27,83^) differences between *S. s. gallaica* and *S. s. bernardezi*, we found extensive gene flow across the entire NG, thus confirming the lack of reproductive isolation between these divergent taxa, and the widespread presence of admixed phenotypes in this contact zone. We found an asymmetric pattern of cyto-nuclear discordances between both lineages. The majority of introgressed individuals exhibited a *S. s. bernardezi* mtDNA, which could suggest a northward expansion by *S. s. gallaica* followed by a mitochondrial capture of *S. s. bernardezi*. This process is potentially in agreement with the male-biased dispersal hypothesis, in which, through population expansions, fire salamander males may disperse longer distances than females^50,84^ and move into their neighbouring subspecies range, thus capturing the local mitochondrial lineage after a few generations. Such a process was suggested as a potential driver of cyto-nuclear discordances among other *S. salamandra* subspecies^31,48,81^. However, *S. s. gallaica* mtDNA reached the northern Galician coast and it is known that *S. s. gallaica* mtDNA is present at low frequencies within the range of *S. s. bernardezi*^38^. Whether this is the result of a recent expansion of both males and females of *S. s. gallaica* into the range of *S. s. bernardezi* or a male-biased colonization of the latter into a historical and wider range of the former remains an open question, with important implications for the mid- and long-term viability of *S. s. bernardezi*, which warrants future research.

In CM, we found very few cases of genetic introgression at both windward and leeward sides of the Cantabrian Mountains, but no signs of hybridization. The negligible signs of admixture and hybridization between the larviparous and large *S. s. bejarae* and the pueriparous and small *S. s. bernardezi* across the CM are likely due to unfavourable environmental conditions of high-elevation areas (see next section). These conditions likely preclude the presence or reduce population densities and activity of fire salamanders, which in turn hinder dispersal across complex topographic areas. However, valleys (1400–1700 m a.s.l.) oriented in a north–south axis might act as mountain passages allowing sporadic dispersal events of fire salamander across the CM.

Like NG, we found high levels of gene flow and hybridization across AC and, consequently, absence of endogenous reproductive barriers between *S. s. bernardezi* and *S. s. fastuosa*. This is an expected result because of their phenotypic similarity and shared recent evolutionary histories. The relatively high proportion of cyto-nuclear discordances found for both subspecies comprises another pattern in common with NG, although the underlying drivers responsible for this genetic pattern are likely different. Selection of traits encoded in the nuclear genome could have boosted such discordances in AC along the expansion wave. Nuclear phylogenomic analyses place *S. s. fastuosa* as the sister taxon of *S. s. bernardezi*, yet *S. s. fastuosa* mtDNA falls within the *S. s. gallaica-bejarae* clade^30^. This genetic discordance fits a demographic scenario where selected traits (i.e. colouration pattern, snout shape and reproductive strategy) encoded by nuclear genes in *S. s. bernardezi* spread eastwards into the *S. s. fastuosa* area during range expansions of favourable climatic cycles in the Pleistocene^31^. It is possible that only the original mitochondrial legacy of *S. s. fastuosa* was maintained though historical mitochondrial capture when both lineages introgressed, a process that has been suggested for *Rana temporaria* in this region^79^. Alternatively, *S. s. fastuosa* could first diverge from *S. s. bernardezi*, and then followed by a process of historical mitochondrial capture of *S. s. gallaica-bejarae*. Further sampling across this region and the use of genomic data and demographic models will allow testing the above contrasting hypotheses.

### Environmental barriers shape gene flow between *S. salamandra* populations

Landscape genetic analyses revealed the IBD model is a strong predictor of genetic differentiation in the NG and AC contact zones and, to a lesser extent, in CM. The marked genetic structure and absence of hybrids across the Cantabrian Mountains suggests this mountain chain is a major barrier to dispersal and gene flow between larviparous and pueriparous lineages, with mountain valleys probably allowing sporadic dispersal events. Interestingly, the observed pattern of genetic differentiation in CM could not be explained by the topographic complexity across this area, as we previously hypothesised, but it was influenced by the climate (IBRclim) and landcover (IBRndvi), albeit with some disagreement between the two genetic distance metrics used here. There is a strong environmental gradient acting between the southern and northern slopes of the Cantabrian Mountains, following the sharp transition between Atlantic and Mediterranean bioclimates, which t is reinforcing the effect that prevents rampant gene flow between *S. s. bernardezi* and *S. s. bejarae* along this CZ (see Supplementary Fig. S1). In addition, the climatic heterogeneity associated with the mountain ridges (e.g. higher wind exposure, colder climate, shorting of the growing season, or rarefaction of surface water bodies) often act as impermeable barriers to fire salamanders^38,85^, other salamander species^86–89^, and anurans^90^. On the other hand, the relatively smooth topography across NG and consequent softer climate transitions in the west–east corridors (i.e. lowlands) across AC likely facilitate salamander dispersal.

Other landscape barriers are likely affecting gene flow across the three studied contact zones. For instance, the reduction and conversion of woodlands to mosaics of agricultural fields, urban settlements, and plantations of exotic trees have been shown to hinder dispersal and enhance genetic differentiation among fire salamander populations^38,41^, though the degree of fragmentation observed in NG and AC appears to be insufficient to prevent hybridization at the spatial scale of this study. Water courses are also known to affect gene flow in *S. Salamandra*, particularly, in *S. s. bernardezi* due to its terrestrial life cycle^91^. Two main rivers (Deva and Nansa) cross the AC hybrid zone in a north–south axis and their locations coincide with a phylogeographic break found in other amphibians^78,79^. Although these rivers might have a role in reducing gene flow between *S. s. bernardezi* and *S. s. fastuosa*, the observed patterns of admixture between these lineages suggest they could act as semi-permeable barriers to gene flow. Also, larviparous individuals would have a greater advantage to overcome these geographic barriers during the larval stage^91^, but our data prevent us from making robust inferences about crossing rates per reproductive mode.

Besides the aforementioned environmental factors, we cannot discount that other phenomena may be at play here. Adaptation to ecological environments has been proposed to explain behavioural (pond vs. stream reproduction^92^, but see^93^) and morphological (colour polymorphism^94^) differentiation in fire salamanders, and it may be a common underlying mechanism explaining the elevated intra-specific phenotypic variation that characterizes this species^27,29,95^. Our study did not identify divergent natural selection (i.e. support for an IBE scenario) as a driver of the distinct morphological and reproductive types across northern Iberian *S. salamandra* populations. Although environmental differentiation observed between southern and northern slopes of the Cantabrian Mountains (i.e. climate and habitat, see Supplementary Fig. S1) resulted in a negligible effect from the IBE model, we cannot discount possible latent adaptive divergence to the different ecological environments found at each side of this mountain chain. We note that the other contact zones also have strong gradients, yet, they occur along the genetic transition. This implies that both subspecies are in contact with the same environmental gradient available in each contact zone. In the Cantabrian Mountains, however, the contact zone coincides with a marked change of the environmental gradient, producing favourable conditions for putative adaptive divergence. These mountains constitute the clearest contact zone where subspecies occupy different environmental niches. While NG and AC are located in a heterogeneous landscape (mosaic of eucalypts plantations, and agricultural bocage in lowland areas, and native forests and scrublands in mid-elevation areas), with a soft change in climatic conditions, in the CM, the wetter northern slopes dominated by Atlantic deciduous forests strongly contrast with the dryer southern slopes exhibiting vegetation typical of Mediterranean ecosystems^96^. Hence, fire salamanders at both sides of the CM likely experience different environmental regimes that may influence their evolutionary trajectories.

The barrier effect imposed by the Cantabrian Mountains, together with a potential ecological adaptation to the local habitat and climate present in the northern and southern slopes of this mountain chain, may also contribute to the reduced levels of admixture and introgression observed between *S. s. bernardezi* and *S. s. bejarae*, which ultimately can lead to speciation events. However, considering the ongoing gene flow between *S. s. bernardezi* and other *S. salamandra* subspecies in environmentally homogeneous contact zones (e.g. NG and AC), our study reinforces the intra-specific polymorphic nature of this species at various phenotypic levels. Finally, we cannot neglect potential historical differences underlying the formation and maintenance of these contact zones. These differences may involve an older and well established hybrid zone in northern Galicia (*S. s. gallaica–S. s. bernardezi*), and a recent secondary contact zone where population edges are still expanding across the CM (*S. s. bejarae*–*S. s. bernardezi*).

## Conclusions

Our study shows that patterns of hybridization between *S. salamandra* subspecies are highly heterogeneous across space, thus emphasizing the role of environmental factors in shaping patterns of hybridization and reproductive isolation. This is best illustrated by the Cantabrian Mountains, which likely comprise an effective barrier to gene flow between the pueriparous *S. s. bernardezi* and larviparous *S. s. bejarae*. Despite the fact that prezygotic and postzygotic barriers were not detected in our study, it is unclear whether hybridization among these subspecies negatively affects survival and fertility of hybrids. This study also has important conservation and taxonomic implications, as the elevation of intra-specific lineages to species level is often based on the reduction of gene flow found between lineages across a small area of their entire contact zone, thus neglecting the environmental heterogeneity, and putative different outcomes of hybridization. Future multidisciplinary studies (integrating phenotypic, behavioural, ecological, and genomic-wide data) are needed to evaluate the potential genomic consequences of hybridization in the fire salamander, the spatio-temporal dynamics of these hybrid zones (i.e. location and spatial extent through time), and the potential role of ecological divergence in the Cantabrian Mountains^9,10,48^.

## Supplementary Information

Supplementary Information.

## Data Availability

The dataset containing microsatellite genotype data, environmental layers, input files, and the R code used to perform optimisation analyses are available upon request. Sequences generated for the mtDNA cytb fragment have been submitted to GenBank.

## References

[1] Abbott RJ, Barton NH, Good JM (2016). Genomics of hybridization and its evolutionary consequences. Mol. Ecol..

[2] Harrison RG, Larson EL (2016). Heterogeneous genome divergence, differential introgression, and the origin and structure of hybrid zones. Mol. Ecol..

[3] Gompert Z, Mandeville EG, Buerkle CA (2017). Analysis of population genomic data from hybrid zones. Annu. Rev. Ecol. Evol. Syst..

[4] Jiggins CD, Mallet J (2000). Bimodal hybrid zones and speciation. Trends Ecol. Evol..

[5] Doebeli M, Dieckmann U (2003). Speciation along environmental gradients. Nature.

[6] Wang IJ, Bradburd GS (2014). Isolation by environment. Mol. Ecol..

[7] Tarroso P, Pereira RJ, Martínez-Freiría F, Godinho R, Brito JC (2014). Hybridization at an ecotone: Ecological and genetic barriers between three Iberian vipers. Mol. Ecol..

[8] Newman CE, Rissler LJ (2011). Phylogeographic analyses of the southern leopard frog: The impact of geography and climate on the distribution of genetic lineages vs. subspecies. Mol. Ecol..

[9] Smith KL, Hale JM, Gay L, Kearney M, Austin JJ, Parris KM, Melville J (2013). Spatio-temporal changes in the structure of an Australian frog hybrid zone: A 40-year perspective. Evolution.

[10] Visser M, Leeuw MD, Zuiderwijk A, Arntzen JW (2017). Stabilization of a salamander moving hybrid zone. Ecol. Evol..

[11] Carneiro M, Baird SJ, Afonso S, Ramirez E, Tarroso P, Teotónio H, Ferrand N (2013). Steep clines within a highly permeable genome across a hybrid zone between two subspecies of the European rabbit. Mol. Ecol..

[12] Gompert Z, Parchman TL, Buerkle CA (2012). Genomics of isolation in hybrids. Philos. Trans. R. Soc. B.

[13] Zieliński P, Dudek K, Arntzen JW, Palomar G, Niedzicka M, Fijarczyk A, Babik W (2019). Differential introgression across newt hybrid zones–evidence from replicated transects. Mol. Ecol..

[14] Hewitt GM (2011). Quaternary phylogeography: The roots of hybrid zones. Genetica.

[15] Naciri Y, Linder HP (2020). The genetics of evolutionary radiations. Biol. Rev..

[16] Kearns AM, Restani M, Szabo I, Schrøder-Nielsen A, Kim JA, Richardson HM, Omland KE (2018). Genomic evidence of speciation reversal in ravens. Nat. Commun..

[17] Butlin R (1987). Speciation by reinforcement. Trends Ecol. Evol..

[18] Arntzen JW, de Vries W, Canestrelli D, Martínez-Solano I (2017). Hybrid zone formation and contrasting outcomes of secondary contact over transects in common toads. Mol. Ecol..

[19] Zamudio KR, Bell RC, Mason NA (2016). Phenotypes in phylogeography: Species’ traits, environmental variation, and vertebrate diversification. Proc. Natl. Acad. Sci..

[20] Devitt TJ, Baird SJ, Moritz C (2011). Asymmetric reproductive isolation between terminal forms of the salamander ring species *Ensatina eschscholtzii* revealed by fine-scale genetic analysis of a hybrid zone. BMC Evol. Biol..

[21] Melo MC, Salazar C, Jiggins CD, Linares M (2009). Assortative mating preferences among hybrids offers a route to hybrid speciation. Evolution.

[22] Cornetti L, Belluardo F, Ghielmi S, Giovine G, Ficetola GF, Bertorelle G, Hauffe HC (2015). Reproductive isolation between oviparous and viviparous lineages of the Eurasian common lizard *Zootoca vivipara* in a contact zone. Biol. J. Linn. Soc..

[23] Rafati N, Blanco-Aguiar JA, Rubin CJ, Sayyab S, Sabatino SJ, Afonso S, Feng C, Alves PC, Villafuerte R, Ferrand N, Andersson L (2018). A genomic map of clinal variation across the European rabbit hybrid zone. Mol. Ecol..

[24] Shipilina D, Serbyn M, Ivanitskii V, Marova I, Backström N (2017). Patterns of genetic, phenotypic, and acoustic variation across a chiffchaff (*Phylloscopus collybita abietinus/tristis*) hybrid zone. Ecol. Evol..

[25] Grabenstein KC, Taylor SA (2018). Breaking barriers: Causes, consequences, and experimental utility of human-mediated hybridization. Trends Ecol. Evol..

[26] Coates DJ, Byrne M, Moritz C (2018). Genetic diversity and conservation units: Dealing with the species-population continuum in the age of genomics. Front. Ecol. Evol..

[27] Velo-Antón G, Santos X, Sanmartín-Villar I, Cordero-Rivera A, Buckley D (2015). Intraspecific variation in clutch size and maternal investment in pueriparous and larviparous *Salamandra salamandra* females. Evol. Ecol..

[28] Beukema W, Nicieza AG, Lourenço A, Velo-Antón G (2016). Colour polymorphism in *Salamandra salamandra* (Amphibia: Urodela), revealed by a lack of genetic and environmental differentiation between distinct phenotypes. J. Zool. Syst. Evol. Res..

[29] Alarcón-Ríos L, Nicieza AG, Kaliontzopoulou A, Buckley D, Velo-Antón G (2020). Evolutionary history and not heterochronic modifications associated with viviparity drive head shape differentiation in a reproductive polymorphic species, *Salamandra salamandra*. Evol. Biol..

[30] Burgon JD, Vences M, Steinfartz S, Bogaerts S, Bonato L, Donaire-Barroso D, Martínez-Solano I, Velo-Antón G, Vieites D, Mable BK, Elmer KR (2021). Phylogenomic inference of species and subspecies diversity in the Palearctic salamander genus *Salamandra*. Mol. Phylogenet. Evol..

[31] García-París M, Alcobendas M, Buckley D, Wake D (2003). Dispersal of viviparity across contact zones in Iberian populations of Fire salamanders (*Salamandra*) inferred from discordance of genetic and morphological traits. Evolution.

[32] Velo-Antón G, García-París M, Galán P, CorderoRivera A (2007). The evolution of viviparity in holocene islands: ecological adaptation versus phylogenetic descent along the transition from aquatic to terrestrial environments. J. Zool. Syst. Evol. Res..

[33] Velo-Antón G, Zamudio KR, Cordero-Rivera A (2012). Genetic drift and rapid evolution of viviparity in insular fire salamanders (*Salamandra salamandra*). Heredity.

[34] Uotila E, Díaz AC, Azkue IS, Rubio Pilarte X (2013). Variation in the reproductive strategies of *Salamandra salamandra* (Linnaeus, 1758) populations in the province of Gipuzkoa (Basque Country). Munibe Cienc. Nat. Nat. Zientziak.

[35] Galán P (2007). Viviparismo y distribución de *Salamandra salamandra bernardezi* en el norte de Galicia. Bol. Asoc. Herpetol. Esp..

[36] Alcobendas M, Dopazo H, Alberch P (1996). Geographic variation in allozymes of populations of *Salamandra salamandra* (Amphibia: Urodela) exhibiting distinct reproductive modes. J. Evol. Biol..

[37] Alarcón-Ríos L, Nicieza AG, Lourenço A, Velo-Antón G (2020). The evolution of pueriparity maintains multiple paternity in a polymorphic viviparous salamander. Sci. Rep..

[38] Lourenço A, Gonçalves J, Carvalho F, Wang IJ, Velo-Antón G (2019). Comparative landscape genetics reveals the evolution of viviparity reduces genetic connectivity in fire salamanders. Mol. Ecol..

[39] Velo-Antón, G., & Buckley, D. Salamandra común—*Salamandra salamandra*. in *Enciclopedia Virtual de los Vertebrados Españoles* (L.M. Carrascal, A Salvador, Eds.) (Museo Nacional de Ciencias Naturales, 2015). Retrieved from http://www.vertebradosibericos.org/anfibios/salsal.html

[40] Cordero A, Velo-Antón G, Galán P (2007). Ecology of amphibians in small coastal Holocene islands: Local adaptations and the effect of exotic tree plantations. Munibe.

[41] Antunes B, Lourenço A, Caeiro-Dias G, Dinis M, Gonçalves H, Martínez-Solano I, Tarroso P, Velo-Antón G (2018). Combining phylogeography and landscape genetics to infer the evolutionary history of a short-range Mediterranean relict, *Salamandra salamandra longirostris*. Conserv. Genet..

[42] Lourenço A, Álvarez D, Wang IJ, Velo-Antón G (2017). Trapped within the city: Integrating demography, time since isolation and population-specific traits to assess the genetic effects of urbanization. Mol. Ecol..

[43] Landguth EL, Cushman SA, Murphy MA, Luikart G (2010). Relationships between migration rates and landscape resistance assessed using individual-based simulations. Mol. Ecol. Resour..

[44] Zhang P, Papenfuss TJ, Wake MH, Qu L, Wake DB (2008). Phylogeny and biogeography of the family Salamandridae (Amphibia: Caudata) inferred from complete mitochondrial genomes. Mol. Phylogenet. Evol..

[45] Hendrix R, Hauswaldt S, Veith M, Steinfartz S (2010). Strong correlation between cross-amplification success and genetic distance across all members of ‘True Salamanders’ (Amphibia: Salamandridae) revealed by *Salamandra salamandra*-specific microsatellite loci. Mol. Ecol. Resour..

[46] Steinfartz S, Kuesters D, Tautz D (2004). Isolation and characterization of polymorphic tetranucleotide microsatellite loci in the Fire salamander *Salamandra salamandra* (Amphibia: Caudata). Mol. Ecol. Notes.

[47] Álvarez D, Lourenço A, Oro D, Velo-Antón G (2015). Assessment of census (N) and effective population size (N e) reveals consistency of N e single-sample estimators and a high N e/N ratio in an urban and isolated population of fire salamanders. Conserv. Genet. Resour..

[48] Antunes, B., Velo-Antón, G., Buckley, D., Pereira, R. & Martínez-Solano, I. Physical and ecological isolation contribute to maintain genetic differentiation between fire salamander subspecies. *Heredity*. 10.1038/s41437-021-00405-0 (2021). 10.1038/s41437-021-00405-0 PMC8102559 33536637

[49] Lourenço A, Sequeira F, Buckley D, Velo-Antón G (2018). Role of colonization history and species-specific traits on contemporary genetic variation of two salamander species in a Holocene island-mainland system. J. Biogeogr..

[50] Lourenço A, Antunes B, Wang IJ, Velo-Antón G (2018). Fine-scale genetic structure in a salamander with two reproductive modes: Does reproductive mode affect dispersal?. Evol. Ecol..

[51] Drummond AJ, Suchard MA, Xie D, Rambaut A (2012). Bayesian phylogenetics with BEAUti and the BEAST 17. Mol. Biol. Evol..

[52] Darriba D, Taboada GL, Doallo R, Posada D (2012). jModelTest 2: More models, new heuristics and parallel computing. Nat. Methods.

[53] Ehl S, Vences M, Veith M (2019). Reconstructing evolution at the community level: A case study on Mediterranean amphibians. Mol. Phylogenet. Evol..

[54] Miller, M. A., Pfeiffer, W., & Schwartz, T. Creating the CIPRES Science Gateway for inference of large phylogenetic trees. in *2010 gateway computing environments workshop (GCE *pp. 1–8) (2010).

[55] Pritchard JK, Stephens M, Donnelly P (2000). Inference of population structure using multilocus genotype data. Genetics.

[56] Jakobsson M, Rosenberg NA (2007). CLUMPP: A cluster matching and permutation program for dealing with label switching and multimodality in analysis of population structure. Bioinformatics.

[57] Jombart T (2008). adegenet: A R package for the multivariate analysis of genetic markers. Bioinformatics.

[58] Anderson EC, Thompson EA (2002). A model-based method for identifying species hybrids using multilocus genetic data. Genetics.

[59] Anderson EC (2008). Bayesian inference of species hybrids using multilocus dominant genetic markers. Philos. Trans. R. Soc. B.

[60] Shurtliff QR, Murphy PJ, Matocq MD (2014). Ecological segregation in a small mammal hybrid zone: Habitat-specific mating opportunities and selection against hybrids restrict gene flow on a fine spatial scale. Evolution.

[61] Shirk AJ, Landguth EL, Cushman SA (2017). A comparison of individual-based genetic distance metrics for landscape genetics. Mol. Ecol. Resour..

[62] Wang J (2011). COANCESTRY: A program for simulating, estimating and analysing relatedness and inbreeding coefficients. Mol. Ecol. Resour..

[63] Queller DC, Goodnight KF (1989). Estimating relatedness using genetic markers. Evolution.

[64] Wang J (2007). Triadic IBD coefficients and applications to estimating pairwise relatedness. Genet. Res..

[65] Estrada-Peña A, Estrada-Sánchez A, de la Fuente J (2014). A global set of Fourier-transformed remotely sensed covariates for the description of abiotic niche in epidemiological studies of tick vector species. Parasit. Vectors.

[66] Karger DN, Conrad O, Böhner J, Kawohl T, Kreft H, Soria-Auza RW, Kessler M (2017). Climatologies at high resolution for the earth’s land surface areas. Sci. Data.

[67] Nosil P, Egan SP, Funk DJ (2008). Heterogeneous genomic differentiation between walking-stick ecotypes: "Isolation by adaptation" and multiple roles for divergent selection. Evolution.

[68] Graves TA, Beier P, Royle JA (2013). Current approaches using genetic distances produce poor estimates of landscape resistance to interindividual dispersal. Mol. Ecol..

[69] Peterman WE, Connette GM, Semlitsch RD, Eggert LS (2014). Ecological resistance surfaces predict fine-scale genetic differentiation in a terrestrial woodland salamander. Mol. Ecol..

[70] Tarroso P, Carvalho SB, Velo-Antón G (2019). Phylin 2.0: Extending the phylogeographical interpolation method to include uncertainty and user-defined distance metrics. Mol. Ecol. Resour..

[71] Peterman WE (2018). ResistanceGA: An R package for the optimization of resistance surfaces using genetic algorithms. Methods Ecol. Evol..

[72] Hutchison DW, Templeton AR (1999). Correlation of pairwise genetic and geographic distance measures: Inferring the relative influences of gene flow and drift on the distribution of genetic variability. Evolution.

[73] Horreo JL, Breedveld MC, Lindtke D, Heulin B, Surget-Groba Y, Fitze PS (2019). Genetic introgression among differentiated clades is lower among clades exhibiting different parity modes. Heredity.

[74] Sota T, Tanabe T (2010). Multiple speciation events in an arthropod with divergent evolution in sexual morphology. Proc. R. Soc. B.

[75] Merrill RM, Van Schooten B, Scott JA, Jiggins CD (2011). Pervasive genetic associations between traits causing reproductive isolation in *Heliconius* butterflies. Proc. R. Soc. B.

[76] Singhal S, Moritz C (2013). Reproductive isolation between phylogeographic lineages scales with divergence. Proc. R. Soc. B.

[77] Donaire D, Rivera XL (2016). salamandra común *Salamandra salamandra* (Linnaeus, 1758) en el subcantábrico: Origen, dispersión, subspecies y zonas de introgresión. Bull. Soc. Catal. Herpetol..

[78] Recuero E, García-París M (2011). Evolutionary history of Lissotriton helveticus: multilocus assessment of ancestral vs. recent colonization of the Iberian Peninsula. Mol. Phylogenet. Evol..

[79] Dufresnes C, Nicieza AG, Litvinchuk SN, Rodrigues N, Jeffries DL, Vences M, Martínez-Solano Í (2020). Are glacial refugia hotspots of speciation and cyto-nuclear discordances? Answers from the genomic phylogeography of Spanish common frogs. Mol. Ecol..

[80] Toews DP, Brelsford A (2012). The biogeography of mitochondrial and nuclear discordance in animals. Mol. Ecol..

[81] Bisconti R, Porretta D, Arduino P, Nascetti G, Canestrelli D (2018). Hybridization and extensive mitochondrial introgression among fire salamanders in peninsular Italy. Sci. Rep..

[82] Dinis M, Merabet K, Martínez-Freiría F, Steinfartz S, Vences M, Burgon JD, Velo-Antón G (2019). Allopatric diversification and evolutionary melting pot in a North African Palearctic relict: The biogeographic history of *Salamandra algira*. Mol. Phylogenet. Evol..

[83] Buckley D, Alcobendas M, García-París M, Wake MH (2007). Heterochrony, cannibalism, and the evolution of viviparity in *Salamandra salamandra*. Evol. Dev..

[84] Helfer V, Broquet T, Fumagalli L (2012). Sex-specific estimates of dispersal show female philopatry and male dispersal in a promiscuous amphibian, the alpine salamander (*Salamandra atra*). Mol. Ecol..

[85] Vörös J, Ursenbacher S, Kiss I, Jelić D, Schweiger S, Szabó K (2017). Increased genetic structuring of isolated *Salamandra salamandra* populations (Caudata: Salamandridae) at the margins of the Carpathian Mountains. J. Zool. Syst. Evol. Res..

[86] Dudaniec RY, Spear SF, Richardson JS, Storfer A (2012). Current and historical drivers of landscape genetic structure differ in core and peripheral salamander populations. PLoS ONE.

[87] Richardson JL (2012). Divergent landscape effects on population connectivity in two co-occurring amphibian species. Mol. Ecol..

[88] Mulder KP, Cortes-Rodriguez N, Campbell Grant EH, Brand A, Fleischer RC (2019). North-facing slopes and elevation shape asymmetric genetic structure in the range-restricted salamander *Plethodon shenandoah*. Ecol. Evol..

[89] Velo-Antón G, Parra JL, Parra-Olea G, Zamudio KR (2013). Tracking climate change in a dispersal-limited species: Reduced spatial and genetic connectivity in a montane salamander. Mol. Ecol..

[90] Sánchez-Montes G, Wang J, Ariño AH, Martínez-Solano Í (2018). Mountains as barriers to gene flow in amphibians: Quantifying the differential effect of a major mountain ridge on the genetic structure of four sympatric species with different life history traits. J. Biogeogr..

[91] Figueiredo-Vázquez, C., Lourenço, A. & Velo-Antón, G. Riverine barriers to gene flow in a salamander with both aquatic and terrestrial reproduction. *Evol Ecol*10.1007/s10682-021-10114-z (2021).

[92] Czypionka T, Goedbloed DJ, Steinfartz S, Nolte AW (2018). Plasticity and evolutionary divergence in gene expression associated with alternative habitat use in larvae of the European Fire Salamander. Mol. Ecol..

[93] Arntzen JW, van Belkom J (2020). ‘Mainland-island’population structure of a terrestrial salamander in a forest-bocage landscape with little evidence for in situ ecological speciation. Sci. Rep..

[94] Burgon JD, Vieites DR, Jacobs A, Weidt SK, Gunter HM, Steinfartz S, Elmer KR (2020). Functional colour genes and signals of selection in colour-polymorphic salamanders. Mol. Ecol..

[95] Velo-Antón G, Cordero-Rivera A (2017). Ethological and phenotypic divergence in insular fire salamanders: Diurnal activity mediated by predation?. Acta Ethol..

[96] González, T. E. D., & Penas, Á. The high mountain area of Northwestern Spain: The Cantabrian Range, the Galician-Leonese Mountains and the Bierzo Trench. In *The vegetation of the Iberian Peninsula* (pp. 251–321). (Springer, 2017).

