# Supplementary material for: Landscape resistance constrains hybridization across contact zones in a reproductively and morphologically polymorphic salamander
Source: Sci Rep. 2021 Apr 29;11:9259. doi: 10.1038/s41598-021-88349-7 (PMC8085075; doi:10.1038/s41598-021-88349-7)

**Supporting information**

**Landscape resistance constrains hybridization across contact zones in a reproductively and morphologically polymorphic salamander**

*Authors*

Guillermo Velo-Antón (GV-A)^1,2*^

André Lourenço (AL)^2,3^

Pedro Galán (PG)^4^

Alfredo Nicieza (AN)^5,6^

Pedro Tarroso (PT)^2^

*Affiliations*

^1^ Universidade de Vigo, Grupo GEA, Departamento de Ecoloxía e Bioloxía Animal, E-36310, Vigo, Spain.

^2^ CIBIO/InBIO, Centro de Investigação em Biodiversidade e Recursos Genéticos da Universidade do Porto. Instituto de Ciências Agrárias de Vairão. R. Padre Armando Quintas. 4485-661 Vairão, Portugal.

^3^ Departamento de Biologia da Faculdade de Ciências da Universidade do Porto. Rua Campo Alegre, 4169-007 Porto, Portugal.

^4^ Grupo de Investigación en Bioloxía Evolutiva (GIBE). Departamento de Bioloxía. Facultade de Ciencias, Universidade da Coruña. Campus da Zapateira, s/n. 15071 – A Coruña, Spain.

^5^ Departamento de Biologıa de Organismos y Sistemas, Universidad de Oviedo,Oviedo, Spain.

^6^ Unidad Mixta de Investigacion en Biodiversidad (UMIB), CSIC-Universidad de Oviedo-Principado de Asturias, Mieres, Spain.

**Corresponding author:**

Velo-Antón G

*E-mail:* guillermo.velo@gmail.com

*ORCID:* https://orcid.org/0000-0002-9483-5695

CIBIO-InBIO, Centro de Investigação em Biodiversidade e Recursos Genéticos da Universidade do Porto

Instituto de Ciências Agrárias de Vairão
R. Padre Armando Quintas 7

4485-661, Vairão. Portugal

**Supplementary tables**

**Table S1** Samples used in this study. This table displays the ID, Sample code, Contact Zone (CZ) and geographic coordinates of each sample.

**Table S2** Optimization results. Two parameters of a logistic function (curvature and inflection point) applied to the isolation by distance matrix are optimized. Original logLikelihood and AIC refer to the GLS model wit non-optimized variables.

**Supplementary figures**

**Figure S1** Maps of the variables used to derive each category (Climate, NDVI and Altitude) for testing landscape genetics models. All analyses on raw variables (PCA for climate, Harmonic regression for NDVI and topographic heterogeneity from altitude; see text for more details) were performed using an extent covering the three contact zones. Areas of contact zones were extracted following the minimum extent of sampling coordinates (in red) plus a ~5 km border. Colour gradients are based on the distribution quantiles of each variable within each contact zone area. Values shown in the scale are the minimum, median and maximum. The dashed white line is the approximate location of each contact zone.

**Figure S2** sPCA results for NG contact zone. Top left: the connection network used to define spatial weightings. Top right: spatial interpolation of the scores of the first eigenvalue of the sPCA. The red contours represent the level of genetic differentiation between samples, being closer when genetic differentiation is high. The grey lines denote regions of abrupt change in allelic frequencies. Middle left: representation of the genetic structure obtained with the first global score showing genetic differentiation between *S. s. bernardezi* (white) and *S. s. gallaica* (black). The size of the squares denotes levels of differentiation (the smaller the square is the less differentiated is the individual). Middle right: this is a variant using grey levels. Bottom: eigenvalues of sPCA with the first eigenvector underlined in black.

**Figure S3** sPCA results for CM contact zone. Top left: the connection network used to define spatial weightings. Top right: spatial interpolation of the scores of the first eigenvalue of the sPCA. The red contours represent the level of genetic differentiation between samples, being closer when genetic differentiation is high. The grey lines denote regions of abrupt change in allelic frequencies. Middle left: representation of the genetic structure obtained with the first global score showing genetic differentiation between *S. s. bernardezi* (white) and *S. s. bejarae* (black). The size of the squares denotes levels of differentiation (the smaller the square is the less differentiated is the individual). Middle right: this is a variant using grey levels. Bottom: eigenvalues of sPCA with the first eigenvector underlined in black.

**Figure S4** sPCA results for AC contact zone. Top left: the connection network used to define spatial weightings. Top right: spatial interpolation of the scores of the first eigenvalue of the sPCA. The red contours represent the level of genetic differentiation between samples, being closer when genetic differentiation is high. The grey lines denote regions of abrupt change in allelic frequencies. Middle left: representation of the genetic structure obtained with the first global score showing genetic differentiation between *S. s. bernardezi* (white) and *S. s. fastuosa* (black). The size of the squares denotes levels of differentiation (the smaller the square is the less differentiated is the individual). Middle right: this is a variant using grey levels. Bottom: eigenvalues of sPCA with the first eigenvector underlined in black.

**Figure S5** Plots showing relationships between the pairwise R_QG_ genetic distance matrix and each pairwise geographic and environmental distance matrix (IBD, IBE_CLIM_, IBE_NDVI_, IBR_ALT_, IBR_CLIM_, IBR_NDVI_) for each contact zone (NG, CM, AC).

**Figure S6** Plots showing relationships between the pairwise R_TRI_ genetic distance matrix and each pairwise geographic and environmental distance matrix (IBD, IBE_CLIM_, IBE_NDVI_, IBR_ALT_, IBR_CLIM_, IBR_NDVI_) for each contact zone (NG, CM, AC).

**Table S1**

| **ID** | **Sample** | **CZ** | **Latitude** | **Longitude** |
| --- | --- | --- | --- | --- |
| 1 | GVA5231 | NG | 43.59 | -8.13 |
| 2 | GVA5229 | NG | 43.61 | -8.12 |
| 3 | GVA5237 | NG | 43.60 | -8.11 |
| 4 | GVA3915 | NG | 43.42 | -8.10 |
| 5 | GVA2953 | NG | 43.41 | -8.08 |
| 6 | GVA3914 | NG | 43.42 | -8.07 |
| 7 | GVA3912 | NG | 43.41 | -8.07 |
| 8 | GVA3913 | NG | 43.41 | -8.07 |
| 9 | GVA5227 | NG | 43.62 | -8.06 |
| 10 | GVA3898 | NG | 43.41 | -8.06 |
| 11 | GVA3899 | NG | 43.41 | -8.06 |
| 12 | GVA3900 | NG | 43.41 | -8.06 |
| 13 | GVA5228 | NG | 43.63 | -8.02 |
| 14 | GVA5216 | NG | 43.50 | -8.01 |
| 15 | GVA5217 | NG | 43.50 | -8.01 |
| 16 | GVA5234 | NG | 43.56 | -8.00 |
| 17 | GVA3909 | NG | 43.45 | -7.99 |
| 18 | GVA3910 | NG | 43.45 | -7.99 |
| 19 | GVA3911 | NG | 43.45 | -7.99 |
| 20 | GVA5242 | NG | 43.45 | -7.99 |
| 21 | GVA3903 | NG | 43.45 | -7.98 |
| 22 | GVA3904 | NG | 43.45 | -7.98 |
| 23 | GVA5249 | NG | 43.71 | -7.98 |
| 24 | GVA5233 | NG | 43.60 | -7.98 |
| 25 | GVA5246 | NG | 43.71 | -7.96 |
| 26 | GVA5245 | NG | 43.72 | -7.95 |
| 27 | GVA5210 | NG | 43.52 | -7.95 |
| 28 | GVA5211 | NG | 43.52 | -7.95 |
| 29 | GVA5223 | NG | 43.61 | -7.94 |
| 30 | GVA5225 | NG | 43.64 | -7.94 |
| 31 | GVA3918 | NG | 43.39 | -7.94 |
| 32 | GVA5219 | NG | 43.49 | -7.93 |
| 33 | GVA5238 | NG | 43.49 | -7.93 |
| 34 | GVA5241 | NG | 43.45 | -7.93 |
| 35 | GVA5218 | NG | 43.55 | -7.92 |
| 36 | GVA5240 | NG | 43.47 | -7.92 |
| 37 | GVA5251 | NG | 43.74 | -7.92 |
| 38 | GVA5250 | NG | 43.74 | -7.92 |
| 39 | GVA5258 | NG | 43.69 | -7.91 |
| 40 | GVA3917 | NG | 43.39 | -7.91 |
| 41 | GVA3916 | NG | 43.39 | -7.91 |
| 42 | GVA3892 | NG | 43.42 | -7.89 |
| 43 | GVA3893 | NG | 43.42 | -7.89 |
| 44 | GVA3894 | NG | 43.42 | -7.89 |
| 45 | GVA5243 | NG | 43.33 | -7.89 |
| 46 | GVA3895 | NG | 43.43 | -7.88 |
| 47 | GVA3902 | NG | 43.52 | -7.88 |
| 48 | GVA3901 | NG | 43.49 | -7.84 |
| 49 | GVA3896 | NG | 43.46 | -7.83 |
| 50 | GVA3897 | NG | 43.46 | -7.83 |
| 51 | GVA3890 | NG | 43.52 | -7.82 |
| 52 | GVA3888 | NG | 43.52 | -7.82 |
| 53 | GVA3889 | NG | 43.52 | -7.82 |
| 54 | GVA3891 | NG | 43.54 | -7.80 |
| 55 | GVA3906 | NG | 43.52 | -7.79 |
| 56 | GVA3844 | NG | 43.43 | -7.75 |
| 57 | GVA3845 | NG | 43.43 | -7.75 |
| 58 | GVA3846 | NG | 43.43 | -7.75 |
| 59 | GVA3847 | NG | 43.43 | -7.75 |
| 60 | GVA3841 | NG | 43.42 | -7.75 |
| 61 | GVA3843 | NG | 43.42 | -7.75 |
| 62 | GVA3848 | NG | 43.44 | -7.73 |
| 63 | GVA3857 | NG | 43.42 | -7.72 |
| 64 | GVA3886 | NG | 43.49 | -7.72 |
| 65 | GVA3887 | NG | 43.49 | -7.72 |
| 66 | GVA3853 | NG | 43.46 | -7.72 |
| 67 | GVA3855 | NG | 43.46 | -7.72 |
| 68 | GVA3856 | NG | 43.46 | -7.72 |
| 69 | GVA3849 | NG | 43.46 | -7.71 |
| 70 | GVA3850 | NG | 43.46 | -7.71 |
| 71 | GVA3851 | NG | 43.46 | -7.71 |
| 72 | GVA3852 | NG | 43.46 | -7.71 |
| 73 | GVA3858 | NG | 43.42 | -7.71 |
| 74 | GVA3854 | NG | 43.45 | -7.70 |
| 75 | GVA5265 | NG | 43.51 | -7.68 |
| 76 | GVA5266 | NG | 43.68 | -7.66 |
| 77 | GVA3859 | NG | 43.40 | -7.66 |
| 78 | GVA2996 | NG | 43.38 | -7.63 |
| 79 | GVA3905 | NG | 43.44 | -7.61 |
| 80 | GVA3908 | NG | 43.49 | -7.59 |
| 81 | GVA3875 | NG | 43.44 | -7.55 |
| 82 | GVA3876 | NG | 43.44 | -7.55 |
| 83 | GVA3870 | NG | 43.42 | -7.54 |
| 84 | GVA3871 | NG | 43.42 | -7.54 |
| 85 | GVA5261 | NG | 43.46 | -7.53 |
| 86 | GVA5262 | NG | 43.46 | -7.53 |
| 87 | GVA3868 | NG | 43.41 | -7.53 |
| 88 | GVA3869 | NG | 43.41 | -7.53 |
| 89 | GVA3872 | NG | 43.42 | -7.53 |
| 90 | GVA3873 | NG | 43.42 | -7.53 |
| 91 | GVA3874 | NG | 43.42 | -7.53 |
| 92 | GVA3865 | NG | 43.40 | -7.52 |
| 93 | GVA3866 | NG | 43.40 | -7.52 |
| 94 | GVA3867 | NG | 43.40 | -7.52 |
| 95 | GVA3877 | NG | 43.45 | -7.52 |
| 96 | GVA3860 | NG | 43.37 | -7.52 |
| 97 | GVA3863 | NG | 43.37 | -7.52 |
| 98 | GVA3864 | NG | 43.37 | -7.52 |
| 99 | GVA3861 | NG | 43.37 | -7.52 |
| 100 | GVA3862 | NG | 43.37 | -7.52 |
| 101 | GVA2982 | NG | 43.53 | -7.50 |
| 102 | GVA2983 | NG | 43.53 | -7.50 |
| 103 | GVA3878 | NG | 43.44 | -7.44 |
| 104 | GVA3879 | NG | 43.44 | -7.44 |
| 105 | GVA3880 | NG | 43.44 | -7.44 |
| 106 | GVA3881 | NG | 43.44 | -7.44 |
| 107 | GVA3882 | NG | 43.45 | -7.43 |
| 108 | GVA3883 | NG | 43.45 | -7.43 |
| 109 | GVA3919 | NG | 43.44 | -7.43 |
| 110 | GVA3927 | NG | 43.44 | -7.43 |
| 111 | GVA3884 | NG | 43.45 | -7.42 |
| 112 | GVA3885 | NG | 43.45 | -7.42 |
| 113 | GVA4258 | NG | 43.40 | -7.40 |
| 114 | GVA4259 | NG | 43.40 | -7.40 |
| 115 | GVA4257 | NG | 43.40 | -7.40 |
| 116 | GVA3922 | NG | 43.31 | -7.39 |
| 117 | GVA3923 | NG | 43.31 | -7.39 |
| 118 | GVA3920 | NG | 43.33 | -7.39 |
| 119 | GVA3921 | NG | 43.33 | -7.39 |
| 120 | GVA3924 | NG | 43.34 | -7.38 |
| 121 | GVA3925 | NG | 43.34 | -7.38 |
| 122 | GVA3926 | NG | 43.34 | -7.38 |
| 123 | GVA3673 | NG | 43.39 | -7.31 |
| 124 | GVA3674 | NG | 43.39 | -7.31 |
| 125 | GVA5267 | NG | 43.40 | -7.23 |
| 126 | GVA5268 | NG | 43.40 | -7.23 |
| 127 | GVA3705 | NG | 43.30 | -7.23 |
| 128 | GVA3026 | CM | 42.77 | -6.67 |
| 129 | GVA3027 | CM | 42.77 | -6.67 |
| 130 | GVA3028 | CM | 42.77 | -6.67 |
| 131 | GVA3029 | CM | 42.77 | -6.67 |
| 132 | GVA3031 | CM | 42.79 | -6.63 |
| 133 | GVA3032 | CM | 42.84 | -6.57 |
| 134 | GVA4505 | CM | 42.98 | -6.56 |
| 135 | GVA4506 | CM | 42.98 | -6.56 |
| 136 | GVA4997 | CM | 42.87 | -6.45 |
| 137 | GVA3574 | CM | 42.91 | -6.36 |
| 138 | GVA3579 | CM | 42.96 | -6.35 |
| 139 | GVA3580 | CM | 42.96 | -6.35 |
| 140 | GVA3581 | CM | 42.96 | -6.35 |
| 141 | GVA3576 | CM | 42.95 | -6.35 |
| 142 | GVA3577 | CM | 42.95 | -6.35 |
| 143 | GVA3578 | CM | 42.95 | -6.35 |
| 144 | GVA3582 | CM | 42.96 | -6.35 |
| 145 | GVA3583 | CM | 42.96 | -6.35 |
| 146 | GVA3584 | CM | 42.96 | -6.35 |
| 147 | GVA3585 | CM | 42.96 | -6.35 |
| 148 | GVA3586 | CM | 42.96 | -6.35 |
| 149 | GVA3587 | CM | 42.97 | -6.34 |
| 150 | GVA3588 | CM | 42.97 | -6.34 |
| 151 | GVA3589 | CM | 42.97 | -6.34 |
| 152 | GVA3590 | CM | 42.97 | -6.34 |
| 153 | GVA3591 | CM | 42.97 | -6.34 |
| 154 | GVA3592 | CM | 42.97 | -6.34 |
| 155 | GVA3612 | CM | 43.09 | -6.34 |
| 156 | GVA3535 | CM | 42.97 | -6.34 |
| 157 | GVA3593 | CM | 42.97 | -6.34 |
| 158 | GVA3594 | CM | 42.97 | -6.34 |
| 159 | GVA3595 | CM | 42.97 | -6.34 |
| 160 | GVA3596 | CM | 42.97 | -6.34 |
| 161 | GVA3597 | CM | 42.97 | -6.34 |
| 162 | GVA3598 | CM | 42.97 | -6.34 |
| 163 | GVA3617 | CM | 43.14 | -6.33 |
| 164 | GVA3613 | CM | 43.10 | -6.33 |
| 165 | GVA3614 | CM | 43.10 | -6.33 |
| 166 | GVA3618 | CM | 43.16 | -6.32 |
| 167 | GVA3609 | CM | 43.17 | -6.31 |
| 168 | GVA3610 | CM | 43.17 | -6.31 |
| 169 | GVA3611 | CM | 43.17 | -6.31 |
| 170 | GVA3608 | CM | 43.17 | -6.29 |
| 171 | GVA3607 | CM | 43.17 | -6.29 |
| 172 | GVA3606 | CM | 43.15 | -6.26 |
| 173 | GVA3602 | CM | 43.09 | -6.25 |
| 174 | GVA3599 | CM | 42.95 | -6.25 |
| 175 | GVA3603 | CM | 43.08 | -6.24 |
| 176 | GVA4989 | CM | 43.11 | -6.24 |
| 177 | GVA4991 | CM | 43.11 | -6.24 |
| 178 | GVA1921 | CM | 43.10 | -6.21 |
| 179 | GVA3600 | CM | 43.00 | -6.19 |
| 180 | GVA3601 | CM | 43.00 | -6.19 |
| 181 | GVA4993 | CM | 43.10 | -6.17 |
| 182 | GVA4999 | CM | 42.97 | -6.13 |
| 183 | GVA3619 | CM | 43.12 | -6.09 |
| 184 | GVA3620 | CM | 43.12 | -6.09 |
| 185 | GVA3621 | CM | 43.12 | -6.09 |
| 186 | GVA4924 | CM | 43.10 | -6.04 |
| 187 | GVA4916 | CM | 43.25 | -6.03 |
| 188 | GVA3624 | CM | 43.16 | -5.98 |
| 189 | GVA3625 | CM | 43.16 | -5.98 |
| 190 | GVA3626 | CM | 43.16 | -5.98 |
| 191 | GVA3532 | CM | 43.03 | -5.91 |
| 192 | GVA3533 | CM | 43.03 | -5.91 |
| 193 | GVA3534 | CM | 43.03 | -5.91 |
| 194 | GVA3628 | CM | 43.23 | -5.87 |
| 195 | GVA3627 | CM | 43.20 | -5.87 |
| 196 | GVA4722 | AC | 43.31 | -5.25 |
| 197 | GVA4723 | AC | 43.31 | -5.25 |
| 198 | GVA4724 | AC | 43.31 | -5.25 |
| 199 | GVA4725 | AC | 43.31 | -5.25 |
| 200 | GVA4726 | AC | 43.31 | -5.25 |
| 201 | GVA4616 | AC | 43.31 | -5.21 |
| 202 | GVA3796 | AC | 43.45 | -5.21 |
| 203 | GVA4642 | AC | 43.44 | -5.19 |
| 204 | GVA4643 | AC | 43.44 | -5.19 |
| 205 | GVA4620 | AC | 43.39 | -5.10 |
| 206 | GVA5119 | AC | 43.44 | -5.09 |
| 207 | GVA5120 | AC | 43.44 | -5.09 |
| 208 | GVA5118 | AC | 43.45 | -5.08 |
| 209 | GVA5102 | AC | 43.44 | -5.07 |
| 210 | GVA5104 | AC | 43.44 | -5.07 |
| 211 | GVA5105 | AC | 43.44 | -5.07 |
| 212 | GVA5106 | AC | 43.44 | -5.07 |
| 213 | GVA5128 | AC | 43.42 | -5.06 |
| 214 | GVA5129 | AC | 43.42 | -5.06 |
| 215 | GVA5130 | AC | 43.42 | -5.06 |
| 216 | GVA3798 | AC | 43.44 | -5.05 |
| 217 | GVA3799 | AC | 43.44 | -5.05 |
| 218 | GVA4667 | AC | 43.40 | -5.03 |
| 219 | GVA4668 | AC | 43.40 | -5.03 |
| 220 | GVA5016 | AC | 43.39 | -5.01 |
| 221 | GVA5017 | AC | 43.39 | -5.01 |
| 222 | GVA5018 | AC | 43.39 | -5.01 |
| 223 | GVA5019 | AC | 43.39 | -5.01 |
| 224 | GVA5020 | AC | 43.39 | -5.01 |
| 225 | GVA4675 | AC | 43.23 | -4.99 |
| 226 | GVA4676 | AC | 43.23 | -4.99 |
| 227 | GVA4610 | AC | 43.27 | -4.98 |
| 228 | GVA4612 | AC | 43.27 | -4.98 |
| 229 | GVA4613 | AC | 43.27 | -4.98 |
| 230 | GVA4614 | AC | 43.27 | -4.98 |
| 231 | GVA5014 | AC | 43.39 | -4.98 |
| 232 | GVA5015 | AC | 43.39 | -4.98 |
| 233 | GVA3102 | AC | 43.40 | -4.98 |
| 234 | GVA5013 | AC | 43.39 | -4.97 |
| 235 | GVA4674 | AC | 43.42 | -4.95 |
| 236 | GVA5070 | AC | 43.43 | -4.94 |
| 237 | GVA5073 | AC | 43.41 | -4.93 |
| 238 | GVA5072 | AC | 43.41 | -4.93 |
| 239 | GVA5071 | AC | 43.41 | -4.93 |
| 240 | GVA5074 | AC | 43.41 | -4.93 |
| 241 | GVA5124 | AC | 43.44 | -4.93 |
| 242 | GVA4692 | AC | 43.36 | -4.92 |
| 243 | GVA4693 | AC | 43.36 | -4.92 |
| 244 | GVA4694 | AC | 43.36 | -4.92 |
| 245 | GVA4695 | AC | 43.36 | -4.92 |
| 246 | GVA4696 | AC | 43.36 | -4.92 |
| 247 | GVA5029 | AC | 43.31 | -4.86 |
| 248 | GVA5023 | AC | 43.42 | -4.85 |
| 249 | GVA5024 | AC | 43.42 | -4.85 |
| 250 | GVA5025 | AC | 43.42 | -4.85 |
| 251 | GVA5026 | AC | 43.42 | -4.85 |
| 252 | GVA5033 | AC | 43.30 | -4.82 |
| 253 | GVA5034 | AC | 43.30 | -4.82 |
| 254 | GVA5035 | AC | 43.30 | -4.82 |
| 255 | GVA4253 | AC | 43.39 | -4.81 |
| 256 | GVA5037 | AC | 43.30 | -4.78 |
| 257 | GVA5038 | AC | 43.30 | -4.78 |
| 258 | GVA5039 | AC | 43.30 | -4.78 |
| 259 | GVA5040 | AC | 43.30 | -4.78 |
| 260 | GVA5032 | AC | 43.39 | -4.74 |
| 261 | GVA4687 | AC | 43.23 | -4.72 |
| 262 | GVA4688 | AC | 43.23 | -4.72 |
| 263 | GVA4689 | AC | 43.23 | -4.72 |
| 264 | GVA4690 | AC | 43.23 | -4.72 |
| 265 | GVA4691 | AC | 43.23 | -4.72 |
| 266 | GVA5041 | AC | 43.32 | -4.72 |
| 267 | GVA5042 | AC | 43.32 | -4.72 |
| 268 | GVA5043 | AC | 43.32 | -4.72 |
| 269 | GVA5044 | AC | 43.32 | -4.72 |
| 270 | GVA5045 | AC | 43.32 | -4.72 |
| 271 | GVA5021 | AC | 43.41 | -4.71 |
| 272 | GVA5022 | AC | 43.41 | -4.71 |
| 273 | GVA5054 | AC | 43.38 | -4.70 |
| 274 | GVA5055 | AC | 43.38 | -4.70 |
| 275 | GVA5056 | AC | 43.38 | -4.70 |
| 276 | GVA5059 | AC | 43.38 | -4.70 |
| 277 | GVA5060 | AC | 43.38 | -4.70 |
| 278 | GVA5061 | AC | 43.38 | -4.70 |
| 279 | GVA4250 | AC | 43.37 | -4.70 |
| 280 | GVA4251 | AC | 43.37 | -4.70 |
| 281 | GVA4252 | AC | 43.37 | -4.70 |
| 282 | GVA4053 | AC | 43.37 | -4.70 |
| 283 | GVA4056 | AC | 43.37 | -4.70 |
| 284 | GVA4057 | AC | 43.37 | -4.70 |
| 285 | GVA5075 | AC | 43.38 | -4.68 |
| 286 | GVA5076 | AC | 43.38 | -4.67 |
| 287 | GVA5030 | AC | 43.38 | -4.67 |
| 288 | GVA5031 | AC | 43.38 | -4.67 |
| 289 | GVA5082 | AC | 43.38 | -4.67 |
| 290 | GVA5083 | AC | 43.38 | -4.67 |
| 291 | GVA5087 | AC | 43.38 | -4.65 |
| 292 | GVA5046 | AC | 43.33 | -4.65 |
| 293 | GVA5047 | AC | 43.33 | -4.65 |
| 294 | GVA5048 | AC | 43.33 | -4.65 |
| 295 | GVA5049 | AC | 43.33 | -4.65 |
| 296 | GVA5095 | AC | 43.38 | -4.64 |
| 297 | GVA5088 | AC | 43.39 | -4.63 |
| 298 | GVA5052 | AC | 43.37 | -4.55 |
| 299 | GVA5053 | AC | 43.37 | -4.55 |
| 300 | GVA5051 | AC | 43.35 | -4.54 |
| 301 | GVA4605 | AC | 43.40 | -4.54 |
| 302 | GVA3801 | AC | 43.37 | -4.50 |

**Table S2**

| **CZ** | **Type** | **Gen. Mat.** | **Original logLik** | **Optimized logLik** | **Original AIC** | **Optimized AIC** | **Delta AIC** | **Curvature** | **Inflection Point** |
| --- | --- | --- | --- | --- | --- | --- | --- | --- | --- |
| NG | IBD | QG | -10747.834 | -10733.979 | 21503.669 | 21475.958 | 27.711 | 3.598 | 0.052 |
| NG | IBD | TRI | -10998.785 | -10966.335 | 22005.570 | 21940.670 | 64.900 | 6.833 | 0.051 |
| CM | IBD | QG | -2912.562 | -2901.405 | 5833.124 | 5810.810 | 22.313 | 3.720 | 0.121 |
| CM | IBD | TRI | -3050.759 | -3003.477 | 6109.517 | 6014.954 | 94.563 | 17.838 | 0.099 |
| AC | IBD | QG | -7490.894 | -7404.659 | 14989.789 | 14817.319 | 172.470 | 7.774 | 0.068 |
| AC | IBD | TRI | -7751.666 | -7607.199 | 15511.332 | 15222.399 | 288.934 | 13.225 | 0.065 |
| NG | IBEclim | QG | -11046.311 | -11036.760 | 22100.622 | 22081.520 | 19.102 | 5.883 | 0.051 |
| NG | IBEndvi | QG | -11198.612 | -11165.250 | 22405.225 | 22338.500 | 66.725 | 51.233 | 0.061 |
| NG | IBEclim | TRI | -11198.714 | -11169.682 | 22405.429 | 22347.364 | 58.065 | 12.936 | 0.050 |
| NG | IBEndvi | TRI | -11311.048 | -11271.590 | 22630.095 | 22551.180 | 78.916 | 69.204 | 0.050 |
| CM | IBEclim | QG | -3042.476 | -3034.301 | 6092.951 | 6076.603 | 16.349 | 998.945 | 0.278 |
| CM | IBEndvi | QG | -3050.594 | -3012.163 | 6109.189 | 6032.325 | 76.863 | 10.413 | 0.285 |
| CM | IBEclim | TRI | -3118.663 | -3102.078 | 6245.326 | 6212.157 | 33.170 | 6.889 | 0.051 |
| CM | IBEndvi | TRI | -3109.533 | -3078.948 | 6227.066 | 6165.896 | 61.170 | 7.912 | 0.144 |
| AC | IBEclim | QG | -7872.546 | -7853.254 | 15753.092 | 15714.509 | 38.583 | 22.649 | 0.051 |
| AC | IBEndvi | QG | -7919.717 | -7882.722 | 15847.435 | 15773.444 | 73.990 | 101.153 | 0.050 |
| AC | IBEclim | TRI | -7978.449 | -7930.420 | 15964.899 | 15868.840 | 96.059 | 44.060 | 0.051 |
| AC | IBEndvi | TRI | -8000.300 | -7957.050 | 16008.601 | 15922.099 | 86.502 | 74.350 | 0.051 |
| NG | IBRclim | QG | -10870.612 | -10869.673 | 21749.225 | 21747.346 | **1.879** | 1.591 | 0.050 |
| NG | IBRndvi | QG | -11211.089 | -11211.089 | 22430.178 | 22430.178 | **0.000** | 494.160 | 0.768 |
| NG | IBRalt | QG | -10808.412 | -10807.981 | 21624.824 | 21623.961 | **0.862** | 1.813 | 0.527 |
| NG | IBRclim | TRI | -11083.344 | -11081.173 | 22174.689 | 22170.345 | **4.344** | 2.340 | 0.060 |
| NG | IBRndvi | TRI | -11323.064 | -11323.064 | 22654.129 | 22654.129 | **0.000** | 396.155 | 0.520 |
| NG | IBRalt | TRI | -11039.971 | -11039.252 | 22087.942 | 22086.504 | **1.439** | 2.396 | 0.197 |
| CM | IBRclim | QG | -2928.153 | -2894.046 | 5864.305 | 5796.092 | 68.214 | 23.436 | 0.200 |
| CM | IBRndvi | QG | -3146.385 | -2893.653 | 6300.769 | 5795.305 | 505.464 | 568.630 | 0.077 |
| CM | IBRalt | QG | -3002.970 | -2948.215 | 6013.939 | 5904.430 | 109.509 | 15.893 | 0.183 |
| CM | IBRclim | TRI | -3042.701 | -3004.437 | 6093.401 | 6016.874 | 76.527 | 16.663 | 0.163 |
| CM | IBRndvi | TRI | -3196.132 | -3066.471 | 6400.264 | 6140.941 | 259.323 | 998.760 | 0.072 |
| CM | IBRalt | TRI | -3106.987 | -3057.457 | 6221.973 | 6122.914 | 99.059 | 11.410 | 0.107 |
| AC | IBRclim | QG | -7942.968 | -7913.069 | 15893.937 | 15834.139 | 59.798 | 999.099 | 0.085 |
| AC | IBRndvi | QG | -7946.313 | -7945.033 | 15900.626 | 15898.066 | **2.560** | 26.074 | 0.051 |
| AC | IBRalt | QG | -7686.175 | -7429.341 | 15380.351 | 14866.682 | 513.669 | 29.431 | 0.112 |
| AC | IBRclim | TRI | -8021.322 | -8013.110 | 16050.643 | 16034.221 | 16.423 | 998.822 | 0.938 |
| AC | IBRndvi | TRI | -8022.820 | -8022.237 | 16053.640 | 16052.473 | **1.166** | 26.146 | 0.050 |
| AC | IBRalt | TRI | -7881.993 | -7676.981 | 15771.987 | 15361.962 | 410.024 | 27.900 | 0.064 |

**Figure S1**


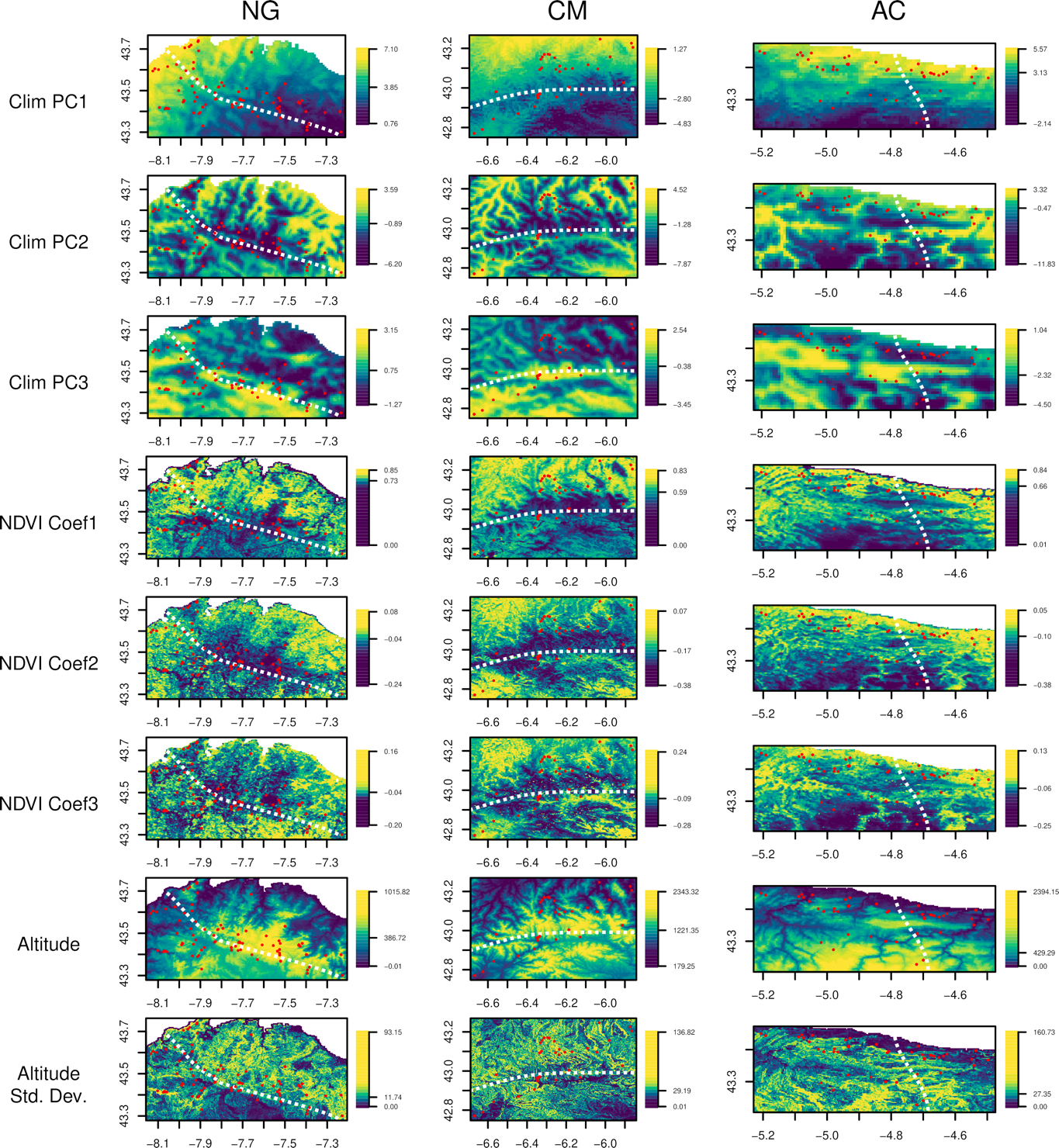


**Figure S2**


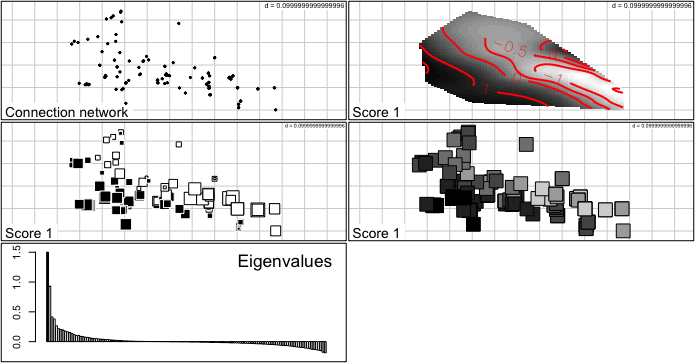


**Figure S3**


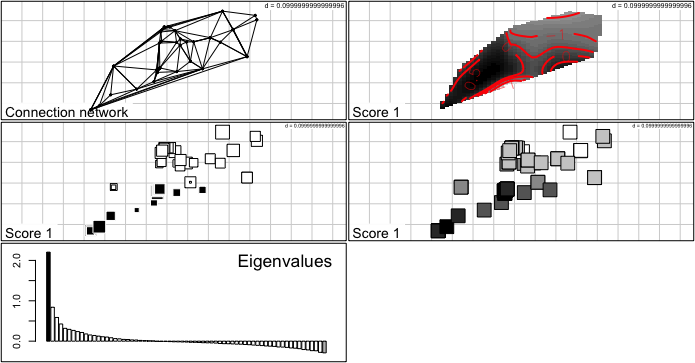


**Figure S4**


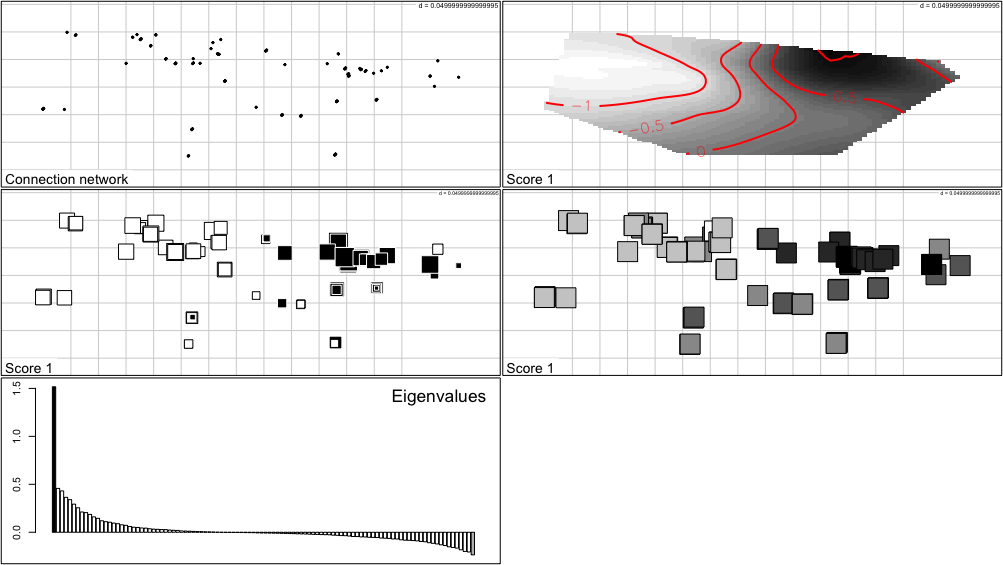


**Figure S5**

**
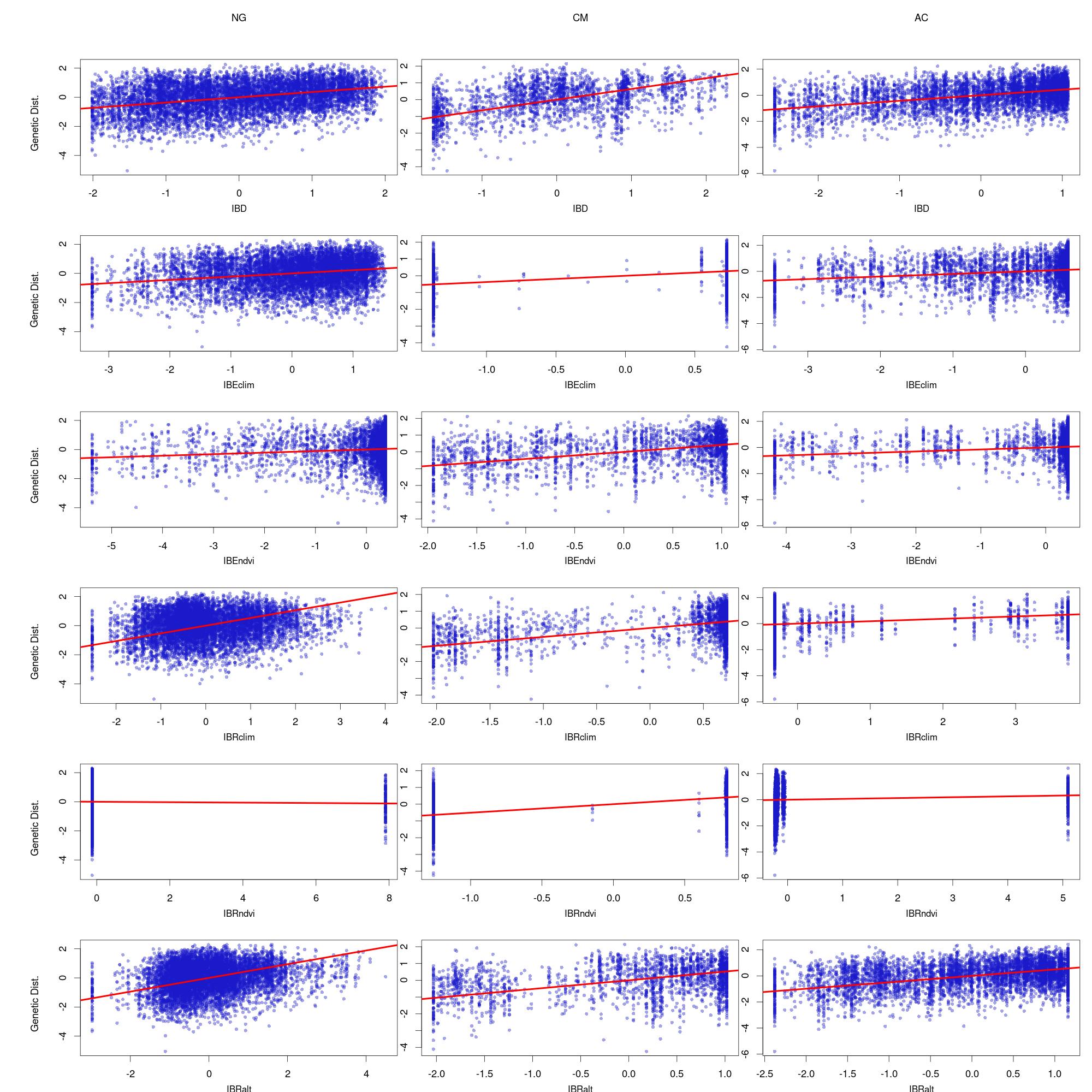
**

**Figure S6**


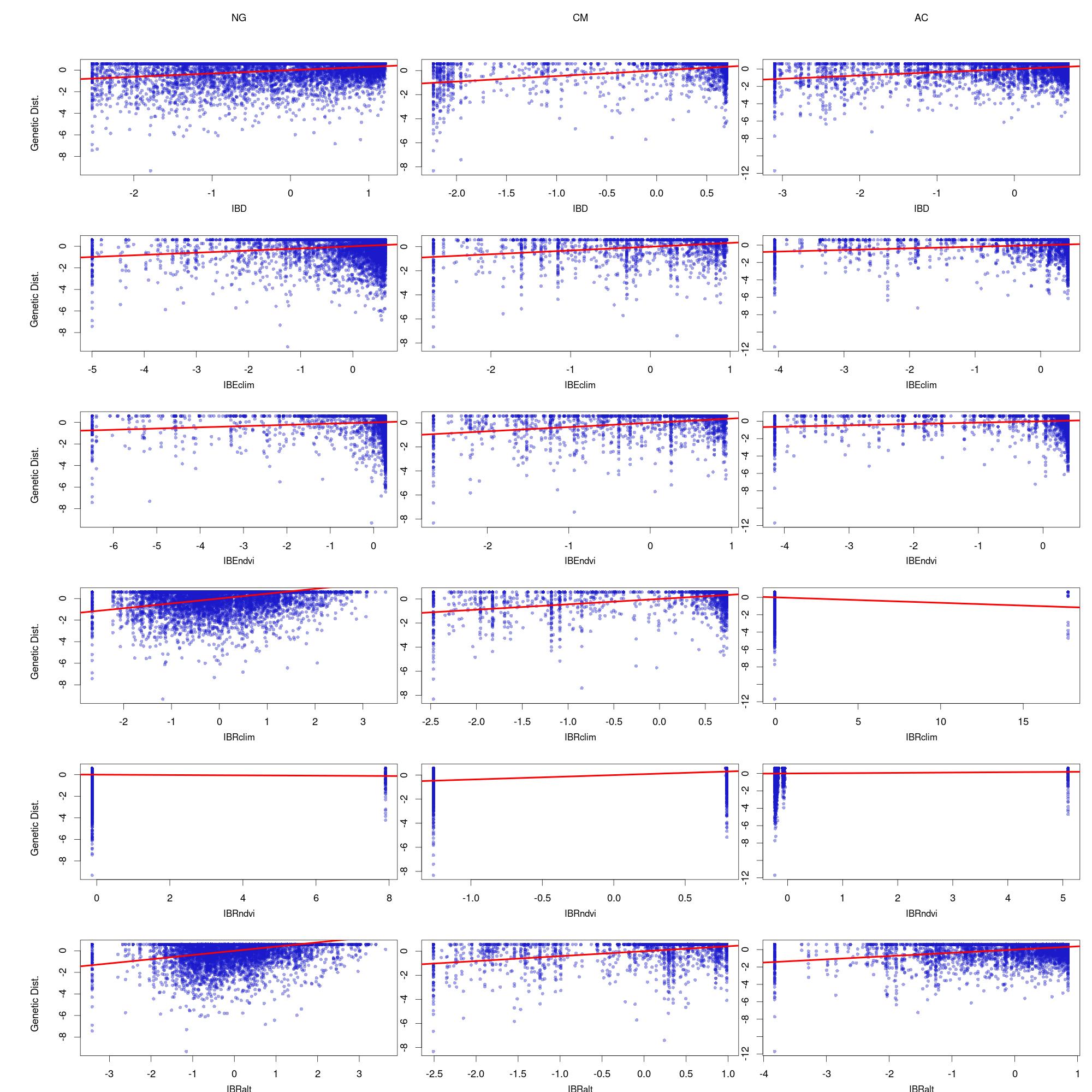

Supplement: Supplementary file 1 — Supplementary Information. [file 41598_2021_88349_MOESM1_ESM.docx]
